# Real estate listings and their usefulness for hedonic regressions

**DOI:** 10.1007/s00181-020-01992-3

**Published:** 2021-01-13

**Authors:** Jens Kolbe, Rainer Schulz, Martin Wersing, Axel Werwatz

**Affiliations:** 1 Chair for Econometrics and Business Statistics, Institute for Economics and Business Law, Technische Universität Berlin, Straße des 17. Juni 135, 10623 Berlin, Germany; 2 University of Aberdeen Business School, Edward Wright Building, Dunbar Street, Aberdeen, AB24 3QY UK

**Keywords:** Hedonic modelling, Nowcasting, Price prediction, Stochastic dominance, C14, C81, R31

## Abstract

Real estate platforms provide a new source of data which has already been used as a substitute for transaction data in hedonic regression applications. This paper asks whether it is valid to do so in the established research areas of (1) willingness to pay estimation, (2) automated valuations, and (3) price index construction. It therefore compares listings and transaction data and regression results derived from them. We find that ask prices stochastically dominate sale prices, mainly because the composition of characteristics differs between the two data sets. But estimates of implicit prices also differ. As a result, willingness to pay estimates from listings data can be widely off when compared with estimates from transaction data. Listings data are not very useful to predict market values of individual houses either, as these predictions suffer from upward bias and large error variance. We find, however, that an ask price index complements a sale price index, as it is useful for nowcasting.

**Supplementary Information:**

The online version contains supplementary material available at 10.1007/s00181-020-01992-3.

## Introduction

The internet is a new source of data for economic research. Such data make it possible to answer research questions that could not be answered before and may make it easier to answer established questions (Edelman 2012). In the area of housing market research, listings from real estate platforms are such a source of new data, which is—contrary to transaction data—readily available. Platforms, such as Immoscout24 (IS24), connect those who plan to sell (customers) with those who intend to buy (users).^1^ This generates an abundance of data, as houses can be listed multiple times until transacted or taken off. Platforms use their own data to attract attention from potential customers and users. IS24, for instance, offers an automated valuation service and publishes the IMX price index. Customers and users might not be aware that these products are based on listings data.^2^

Economists have started to use the timely listings data as a *substitute* for transaction data in such established areas of housing market research as hedonic pricing, automated valuation, and index construction. In *hedonic pricing* applications, the willingness to pay (WTP) for non-traded amenities is estimated with regressions of price on characteristics. The estimates can then be used to assess public policies, such as a ban on night flights (Taylor 2017). Bauer et al. (2017), Kholodilin et al. (2017), and Winke (2017) use listings data to estimate WTP for nuclear power plant closures, energy efficiency of buildings, and aircraft noise reduction, respectively. In *automated valuation* applications, market values of properties are predicted based on fitted regression models. Financial institutions use these valuations, for instance in the process of mortgage securitisation. While the regressions are usually fit with transaction data, Antipov and Pokryshevskaya (2012) and Pérez-Rave et al. (2019) use listings data instead. In *index construction* applications, price trends of average properties should be measured over time. As properties are heterogeneous, regressions are used to control for property heterogeneity. Central banks need price indices to guide policy and financial institutions need them for loan portfolio risk management. Arguably, ask price indices can be useful in this context. Bauer et al. (2013), in work that informed the IMX of IS24, go further and argue that price trends can be measured better and at higher frequency with abundant listings than with sparse transaction data.

In this paper, we examine whether it is valid to use listings as substitute for transaction data in the three applications of housing market research. We approach this question in three steps. In the first step, presented below, we review the literature on the role of listings in the selling process and ask whether one should expect listings to be a valid substitute for transactions, the outcome of this process. The second step and third step use listings and transaction data of single-family houses from Berlin, Germany, to examine empirically the question in detail by comparing the data sets and the regressions results derived from them.

The literature on the house selling process raises doubts that listings data are a valid substitute for information on actual transactions. According to the literature, a rational seller will use the ask price to attract interest and signal which bid will be accepted with certainty, but bids below the ask price will also be accepted if higher than a seller’s private reservation value. The ask price is therefore an upper bound for the sale price if the listing is successful. Horowitz (1992) derives such seller behaviour in a simple framework, and Yavaş and Yang (1995) extend the framework by introducing brokers working on behalf of sellers. The extension by Chen and Rosenthal (1996) is more substantial, as they introduce uncertainty for potential buyers who must learn first whether a house is suitable. Inspecting a house is costly, even more so as an opportunistic seller will try to appropriate buyer’s surplus if the house is suitable. A public ask price limits the inspection cost for potential buyers as it commits the seller. Chen and Rosenthal (1996) also examine bargaining and find that the seller can counter bargaining power of the buyer up to a point. Only a weak seller will not bargain and sell at the ask price, see also Arnold (1999). We see that successful listings have an ask price higher than the sale price. All else equal, unsuccessful listings will have an even higher ask price. This conjecture is supported by empirical evidence from the literature.^3^

If the markup does not vary across properties, it will not bias the relationship between prices and characteristics. It would be absorbed simply by the constant term in regression applications. However, as the selling process involves bargaining between heterogeneous and differently informed sellers and buyers, a fixed mark-up seems implausible, see Merlo and Ortalo-Magné (2004). Obviously, if a seller who is good at haggling meets a buyer who is not, we expect a higher transaction price than otherwise, all else equal. Genesove and Mayer (1997) present evidence that sellers who have mortgage loans with high LTVs hold out for longer and sell at higher prices (if they can sell). Harding et al. (2003) find evidence that seller and buyer characteristics impact on the sale price. Harding et al. (2003) find some evidence that seller and buyer characteristics should be included in regression applications to prevent biased estimates of hedonic prices. As seller and buyer have been matched in a transaction, their characteristics, if observed, can be used to control for effects of bargaining. Such control can never be implemented with listings data, as it is not known whether a house will be sold, to whom, and at which price.^4^ Successful bargaining should also result in an improved description of the house. In a listing, a seller might not report characteristics that make a house unattractive or misreport characteristics in error. A diligent buyer will ensure that all information on the property is recorded correctly and this will show up in the transaction data. The literature reviewed so far shows that ask prices and characteristics may have a different relationship than sale prices and characteristics, because listings data may lack relevant house characteristics and has not gone through the process of successful bargaining.

A recent strand of the literature uses insight from behavioural economics to understand how ask prices are set. Genesove and Mayer (2001) assume that sellers are prone to loss aversion. They find that sellers who experienced nominal losses since the purchase of the property they want to sell, set high ask prices and are willing to wait for a long time for a good offer. If such an offer does not arrive, the house will eventually be taken off the market. This is relevant here for two reasons. First, listings data may contain many observations with comparatively high ask prices and low transaction probabilities. Second, ask prices might be backward looking. This should not only have an impact on the relative pricing of characteristics, but Genesove and Mayer find also evidence that ask prices take several quarters to adjust to market prices. This questions whether price indices based on ask prices can serve as nowcasts for transaction price indices that are only observed with delay.

The literature review indicates that listings data and transaction data are different and that it is unlikely that the former can substitute for the latter. In the rest of the paper, we analyse the distributions of our listings and transaction data and, as we find that the distributions differ, examine possible reasons for the selectivity of the listings data. We use then the listings and transaction data for the regression applications of hedonic pricing, automated valuation, and index construction and examine what the selectivity implies in economic terms.

Our main empirical findings are as follows. First, ask and sale prices are distributed differently, mainly because the composition of characteristics differs. This complements the finding of Shimizu et al. (2016) for condominium data from Tokyo. We go further and establish that characteristics in the listings data are stochastically larger than their counterparts in the transaction data. We explore also possible explanations of *why* the characteristics distributions differ as they do. We find some evidence that platforms attract more sophisticated customers and users. Second, we obtain the new finding that estimates of implicit prices differ statistically between the two data sets and that this difference enforces the distributional dominance relationship. Third, we obtain that regression applications fitted to listings data can give implicit price functions that are counterintuitive. To impose as little structure as possible in these applications, we use semiparametric additive models. Estimates of average WTP can also be widely off when compared with the estimates from transaction data. Listings data are not very useful to predict market values of individual houses either, as these predictions suffer from upward bias and large error variance. We also find that an ask price index is not a substitute for a sale price index but a complement, as we obtain statistical evidence that it is useful for nowcasting.

Our results are in line with the literature on self-assessed property market values. While self-assessed market values are not the same as ask prices, both are *before* any contact with potential buyers. The common evidence shows that owners overestimate market value, see Goodman and Ittner (1992) and Kiel and Zabel (1999). Banzhaf and Farooque (2013) find that self-assessed values are less useful in hedonic analysis than transaction prices. The recent paper by Bigelow et al. (2020) contains an excellent summary of this literature. Their study compares hedonic prices estimated from self-assessed values and market transactions and finds that owners misjudge the value of land characteristics that are salient to them. Consequently, hedonic prices for such characteristics estimated with self-assessed values differ from those estimated with transaction prices.

The rest of the paper is organised as follows. Section 2 describes and analyses the transaction and listings data. Section 3 explains the implementation of the three regression applications and presents results. Section 4 concludes. The web-appendix provides further details.

## Transaction and listings data-description and analysis

### Data sources

The data cover the period 2007–2015. The transaction data are provided by Berlin’s surveyor commission (GAA, Gutachterausschuss für Grundstückswerte in Berlin). By law, surveyor commissions are obliged to keep a detailed record of each and every real estate transaction that takes place in Berlin. To facilitate this, commissions have access to sale contracts, administrative data, and can request further clarification from parties involved in a transaction. Each observation has information on the sale price, physical and legal characteristics of the building and the plot, such as rights of way, and whether the buyer or seller is a public or private legal entity, such as a housing association or a real estate fund. We have also information on the legal specifics of transactions, such as personal or business relationships between the contracting parties, such as divorce and inheritance or a sale that stipulates deferred payment. As such transactions are not arm’s-length, we do not use them. This leaves us with 17,650 observations of market transactions in the GAA data.

The listings data are provided by IS24, the largest real estate platform in Germany.^5^ IS24 brings not only customers (potential sellers) and users (potential buyers) in contact, but allows also third parties, such as agents, mortgage banks, and appraisers, to advertise their services. IS24 listings are similar to classified ads, but modern technology gives much more flexibility. For instance, the customer can modify the content of an ad during a listing’s term; it is also possible to extend the term, while the listing is still active.^6^ Users searching for properties can register with IS24 and will receive afterwards personalised newsletters with updates on visited listings and links to similar properties on offer. As marketing platform, IS24 takes no responsibility that the information on listed properties is complete, correct, and that the properties are still available, i.e. have not been sold in the meantime (IS24 Terms and Conditions, Immobilien Scout 24 (2018, 5.1, 6.1, 9.1)).

For each IS24 listing, we keep only the information from the last day for which it is observed. *If* the listing was successful and a buyer could be found, then this is the date closest to the transaction. Obviously, a listing could also have ended without having attracted a buyer. The property might be listed later again under a different identification code, perhaps with slightly varied information on the property. It is also possible that the very same property is marketed by different agents separately. Furthermore, developers use platforms to advertise different specifications of new projects before going ahead to learn which ones find most interest. These aspects motivate why the IS24 data have 144,274 observations, about 8.2 times as many as the GAA data.

### Data cleaning

The IS24 data suffer from many patchy observations, the result of relying solely upon customer provided information. We concentrate on observations with sale (GAA) and ask (IS24) price that have complete entries for the following *core* characteristics: plot area, floor area, building age, house type, and administrative district in which the house is located.^7^ In some parts of our examination, we use coordinates to model location values.

Despite the fairly small set of core characteristics, Table 1 shows that 26% of observations in the IS24 data must be removed. No observation in the GAA data must be removed, a sign of data quality.

**Table 1:** Effects of data cleaning. Gives the number of observations in the original data and after each step of the data cleaning procedure. Missing values refer to observations that lack entries for some of the core variables. Old refers to a house that has a building which is older than 100 years at the date of transaction or the last listing day. Bounds for plot area, floor area, and transaction price per floor area come from annual reports of the GAA

	GAA	IS24
Original data	17,650	144,274
After removing		
Missing values	17,650	106,193
Old or under construction	15,242	83,952
Outwith bounds	12,524	68,070

The remaining rows in Table 1 show the effects of deleting unusual observations. First, we remove observations of development projects and houses that are either still under construction or older than 100 years. Both are different from standard houses in the sense that the former do not exist yet and that the latter have existed for longer than usual. This reduces the number of observations by 14% (GAA) and 21% (IS24). Second, we apply bounds to the plot area, the floor area, and the price to floor area ratio. A researcher equipped only with listings data would use such publicly available information for data preparation.^8^ We treat the GAA data equally and apply the same bounds to it. This reduces the numbers of observations by 18% (GAA) and 19% (IS24). The final data sets have 12,524 (GAA) and 68,070 (IS24) observations; we refer to the former as *sale* (index *s*) and the latter as *ask* data (index *a*).

### Comparative analysis of the two data sets

We start the analysis with a close examination of the two data sets. Table 2 presents descriptive statistics of prices and characteristics. On average, houses in the ask data have higher prices, are younger and have larger floor and plot areas than houses in the sale data. Given the literature, higher ask prices are to be expected and Shimizu et al. (2016) also observe differences in characteristics.

**Table 2:** Summary statistics for sale and ask data sets. Panel A gives also information for variables other than the core variables. Prices are in 000’ Euros. Age of building at the date of sale or end of listing, respectively. Floor and plot area are in sqm. Legal entity indicates that buyer (seller) is an housing associations, real estate fund, or other legal entity

	Mean	SD	Min	Max
*Panel A. Sale data* ($N=12,524$)				
Price ($P_s$)	250.50	129.07	40.00	1450.00
$\ln$ Price ($p_s$)	12.33	0.45	10.60	14.19
Age	44.94	30.37	0.00	100.00
Floor area	144.78	52.79	41.00	642.00
Plot area	544.96	267.04	112.00	1500.00
Detached	0.56			
Semi-detached	0.28			
Terraced house	0.17			
Listed building	0.05			
Prefabricated	0.10			
Converted attic	0.53			
Swimming pool	0.01			
Flat roof	0.15			
No basement	0.18			
Backland development	0.17			
Lake/river access	0.01			
Condition of building				
Poor	0.04			
Average	0.62			
Good	0.34			
Neighbourhood amenity rating				
Poor	0.30			
Average	0.51			
Good	0.18			
Excellent	0.01			
Legal entity				
Buyer	0.02			
Seller	0.20			
*Panel B. Ask data* ($N=68,070$)				
Price ($P_a$)	320.96	189.96	45.00	2020.00
$\ln$ Price ($p_a$)	12.56	0.46	10.71	14.52
Age	32.02	27.92	0.00	100.00
Floor area	187.21	74.93	50.00	650.00
Plot area	583.49	267.37	100.00	1500.00
Detached	0.68			
Semi-detached	0.23			
Terraced house	0.09			

**Fig. 1:**
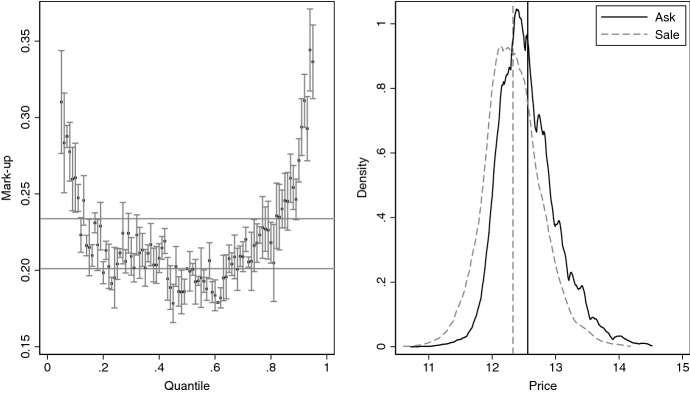
Distributions of ask and sale prices. Left panel shows markups of ask over sales prices at different quantiles. Horizontal lines give markups at the medians (20.1%) and means (23.4%). Whiskers give pointwise confidence intervals at the 0.95 level. Right panel shows kernel density estimates of the distributions of prices from ask data set (solid black) and from sale data set (dashed gray). Bandwidths are chosen with Silverman’s (1986) rule-of-thumb. Vertical lines are the respective means of the ask and sale prices

The markup of ask to sale price is 28% for the arithmetic averages (${\overline{P}}_{a}/{\overline{P}}_{s}-1)$ and 26% for the geometric averages ($\exp \{{\overline{p}}_a-{\overline{p}}_s\}-1$). The markups are sizeable and similar to those reported in Shimizu et al. (2016). Figure 1 gives further evidence on the price distributions, where we concentrate on log prices, as it is common in the literature. The left panel shows the markups for the percentiles of the price distributions.^9^ The markups are particularly high in the tails. All markups are strictly positive and statistically significant. Given the density estimates in the right panel, it seems that ask prices dominate sale prices stochastically, which would imply $F_a(p)-F_s(p) \leqslant 0$ for all $p\in [0,\max (p_a,p_s)]$.^10^ The dominance is strong if the inequality is strict for some *p*.

We test this with the Kolmogorov–Smirnov (KS) statistic ($j\ne k$)

1 $$\begin{aligned} {\widehat{d}}_{j,k} = \left( \frac{N_jN_k}{N_j+N_k}\right) ^{0.5}\sup _p \left\{ {\widehat{F}}_{j}(p) - {\widehat{F}}_{k}(p) \right\} \end{aligned}$$

and the procedure of Barrett and Donald (2003, p. 75). Hats denote estimators and $N_i$ the number of observations. The null hypothesis is $F_j(p)-F_k(p)\leqslant 0$ over the full support and the test statistic focuses on the most unfavourable outcome for the null. If the null is true, we expect ${\widehat{d}}_{j,k}\leqslant 0$. If the alternative $F_j(p)>F_k(p)$ is true for at least one *p*, we expect ${\widehat{d}}_{j,k}>0$. The procedure works as follows. First, we test whether ${\widehat{d}}_{a,s}\leqslant 0$. If we cannot reject, we continue and test whether we can reject ${\widehat{d}}_{s,a}\leqslant 0$. If we can reject, we have established strong dominance. Table 3 presents the statistics for the price distribution in Panel A. We conclude that ask prices dominate sale prices strictly at all of the usual significance levels (0.001, 0.01, 0.05). This implies also that $\text{ E }[{p}_a]>\text{ E }[{p}_s]$, whereas the reverse does not necessarily apply. It has been observed before in the literature that ${\overline{p}}_a>{\overline{p}}_s$, but our evidence on the price distributions is much stronger.

**Table 3:** Stochastic dominance tests. The statistic ${\widehat{d}}_{j,k}$ ($j\ne k$) tests the null hypothesis that distribution *j* dominates distribution *k* weakly. In Panel A, the variable tested for is $-AGE$, ${\widehat{d}}_{j,k}$ is the signed two sample KS test statistic, defined in Eq. (1). The *p* values for the null are calculated as $\exp \{-2({\widehat{d}}_{j,k})^2\}$, see Barrett and Donald (2003, p. 78). In Panel B, ${\widehat{d}}_{j,k}$ is the KS maximal *t*-statistic as defined in Chernozhukov et al. (2013, p. 2222). The *p*-values for the null are calculated as $R^{-1}\sum _r 1({\widehat{d}}_{j,k,r}^{*}>{\widehat{d}}_{j,k})$, where ${\widehat{d}}_{j,k,r}^{*}$ is the $r'th$ bootstrap test statistic, see Barrett and Donald (2003, p. 8). The number of bootstrap replications is 200

	${\widehat{d}}_{a,s}$	*p* value	${\widehat{d}}_{s,a}$	*p*-value
*Panel A. Marginal distributions*				
Price	0.000	1.000	20.577	0.000
Age	0.286	0.849	24.689	0.000
Floor area	0.000	1.000	34.102	0.000
Plot area	0.027	0.999	9.007	0.000
*Panel B. Price decomposition*				
Price	0.000	0.915	48.777	0.000
Characteristics	0.000	0.860	53.132	0.000
Implicit prices	1.076	0.705	10.978	0.000

**Fig. 2:**
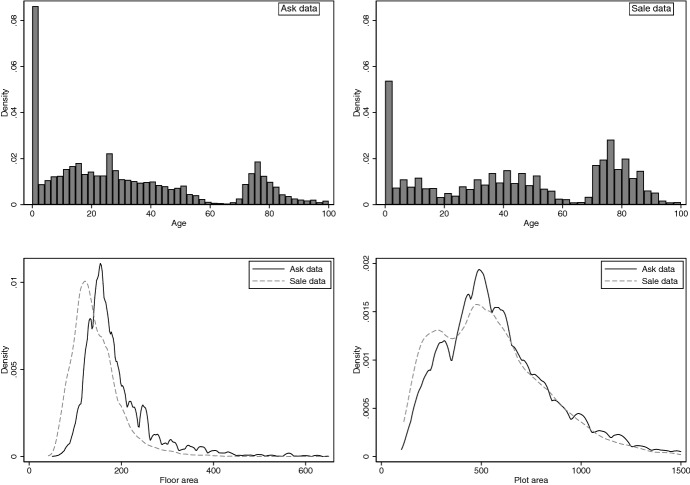
Distributions of house characteristics. Top panel shows histograms of building age for the observations in the ask and the sale data, respectively. Lower panel shows kernel density estimates of floor area (left) and plot area (right) for the observations in the data. Bandwidths are chosen with Silverman’s (1986) rule-of-thumb

The strong dominance of the ask price distribution could be caused *either* because the house characteristics differ between the data sets *or* because the characteristics are valued differently or because *both* effects play a role. We explore these issues by comparing the distribution of characteristics in the two data sets, followed by a decomposition analysis. The estimates in Fig. 2 indicate that houses in the ask data not only have different averages of age, floor area and plot area, but that characteristics in the ask data dominate those in the sale data. This is confirmed by the results of strong dominance tests reported in Panel A of Table 3.^11^

**Fig. 3:**
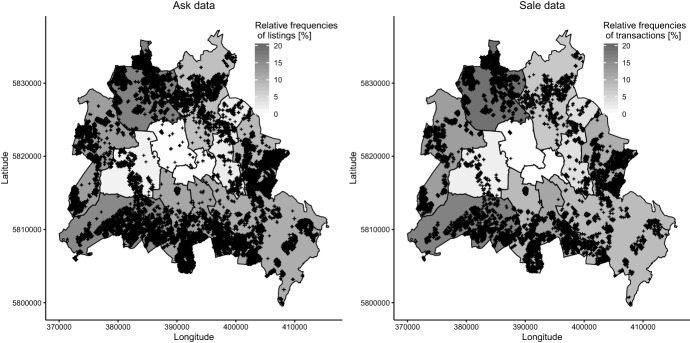
Spatial distribution of ask and sale observations. Shows the relative frequency of observations in the ask and sales data across Berlin’s 12 administrative districts. Crosses give locations of the 59,502 (12,218) individual observations in the ask (sale) data for which we have coordinates

Table 2 shows that the proportion of detached houses is higher in the ask than in the sale data and the proportion of terraced houses is lower.^12^ This could explain why the floor and plot area distributions of the ask data dominate those of the sale data. Figure 3 shows that the observations of the two data sets follow a similar spatial cluster and are, at the level of Berlin’s administrative districts, nearly identical with a correlation of $\rho =0.97$. The observed characteristics can therefore not explain why the houses in the ask data are dominantly younger.

In summary, the analysis of the core characteristics age, floor and plot area shows strong dominance of observations in the ask over their counterparts in the sale data. We explore next whether this is the sole reason for the strong dominance of ask over sale prices.

### Decomposition analysis

We use the inference procedure of Chernozhukov et al. (2013) to assess the importance of house characteristics for the differential of ask and sale price distributions. The procedure is similar in spirit to the decomposition at the means of Blinder (1973) and Oaxaca (1973). The starting point is the following formula which—akin the law of iterated expectations—relates unconditional and conditional distribution functions

2 $$\begin{aligned} F_{j|k}(p) \equiv \int _{{\mathscr {X}}_k} F_{P_{j}|X_{j}}(p|\mathbf {x}) d F_{X_k}(\mathbf {x}) \end{aligned}$$

$F_{P_{i}|X_i}(p|\mathbf {x})$ is the price distribution conditional on the vector $\mathbf {x}$ of characteristics and $F_{X_i}(\mathbf {x})$ is the distribution of these characteristics. If $k=j$, both the conditional price distribution and the distribution of characteristics come from the same data set and Eq. (2) reduces to the unconditional price distribution function $F_{j|j}(p)=F_j(p)$. For $k\ne j$, however, Eq. (2) considers counterfactual situations. For instance, combining the conditional price distribution $F_{P_{a}|X_{a}}(p|\mathbf {x})$ of the ask data with the distribution of characteristics $F_{X_s}(\mathbf {x})$ of the sale data, leads to the counterfactual distribution $F_{a|s}(p)$. This is the distribution of ask prices that would prevail if the actual relationship between ask prices and characteristics were combined with the distribution of characteristics found in the sale data. The counterfactual distribution allows to decompose of the difference between the ask and sale price distributions

3 $$\begin{aligned} F_a(p) - F_s(p)=\{F_{a|a}(p)-F_{a|s}(p)\}+\{F_{a|s}(p)-F_{s|s}(p)\} \end{aligned}$$

The first term on the right-hand side of Eq. (3) reflects differences due to the composition of *characteristics* in the data and the second term reflects differences due to different relationships between prices and characteristics in both groups. From the viewpoint of hedonic regression, the second component quantifies the component of $F_a(p) - F_s(p)$ due to differences in *implicit pricing* of these characteristics among listed and transacted houses.

To test whether each of the two terms on the right-hand side of Eq. (3) obeys a stochastic dominance relationship, we proceed as follows.^13^ First, we estimate the distribution functions with

4 $$\begin{aligned} {\widehat{F}}_{j|k} (p)= & {} c + \frac{1-2c}{(G-1)N_k} \sum _{n=1}^{N_k} \sum _{g=1}^{G} \mathbf {1}\left( \mathbf {x}_{k,n}' \widehat{\varvec{\beta }}_j(\tau _g) \leqslant p \right) \end{aligned}$$

The argument of the indicator function $\mathbf {1}(\cdot )$ is the characteristics bundle of observation *n* from data set *k* evaluated at implicit prices $\widehat{\varvec{\beta }}_{j}(\tau _g)$ estimated with a quantile regression with all observations from data set *j*. In particular, we regress the price on third degree polynomials of the continuous core characteristics, and on house type, district, and yearly time dummies.^14^ Second, we use KS statistics and bootstrapped *p*-values to test for dominance in the terms of Eq. (3).

Starting with $F_a(p) - F_s(p),$ the results in the first row of Panel B of Table 3 show that ask prices dominate sale prices as before at the usual significance levels. The slightly different KS test statistics and *p*-values result from the price distributions now being estimated with Eq. (4) instead of with the raw data. Turning to the role of characteristics therein, we find that $F_{a|a}(p)-F_{a|s}(p)\leqslant 0$ at all the usual significance levels. That is, when evaluated at the same implicit prices, the characteristics in the ask data strongly dominate those in the sale data. This was to be expected from the stochastic dominance results for the continuous house characteristics of Panel A. We also find that—once the characteristics are accounted for—the pricing of characteristics in the ask data strongly dominates those in the sale data, i.e. $F_{a|s}(p)-F_{s|s}(p)\leqslant 0$, at all the usual significance levels. Hence, in all, prices in the ask data dominate those in the sale data both with respect to characteristics *and* implicit prices.

To quantify the contribution of house characteristics and implicit prices to the ask price markups across the distribution (see Fig. 1), we use the quantile version of Eq. (3)

5 $$\begin{aligned} Q_a(\tau ) - Q_s(\tau ) = \left\{ Q_{a|a}(\tau ) - Q_{a|s}(\tau ) \right\} + \left\{ Q_{a|s}(\tau ) - Q_{s|s}(\tau ) \right\} \end{aligned}$$

where $Q_{j|k}(\tau )$ is the $\tau$th quantile of the distribution of the price in data set *j*, given the characteristics in data set *k*. We obtain the quantiles by inverting the estimated distribution functions ${\hat{F}}_{j|k}(p)$. To conduct inference, we compute bootstrap pointwise and uniform confidence bands. Results of the decompositions at the mean and for nine quantiles are reported in Table 4.^15^

**Table 4:** Decomposition of markups. Shows decomposition of the ask and sale price distributions. Standard errors for the mean (quantile) decomposition are computed using the Huber-White covariance estimator (bootstrapped interquartile range of ${\hat{F}}_{j|k}(p)$). Pointwise confidence intervals use critical values from *N*(0, 1). Uniform confidence bands use empirical quantile of bootstrapped KS maximal *t*-statistic, see Chernozhukov et al. (2013, p. 2222). The number of bootstrap replications is 200. Confidence level is set to 0.95

	Estimated	Standard	Pointwise		Uniform	
	Effect	Error	Conf. Interv.		Conf. Bands	
*Panel A. Markup*						
Mean Quantile	0.234	0.004	0.225	0.242		
0.1	0.241	0.006	0.230	0.253	0.223	0.260
0.2	0.210	0.005	0.201	0.219	0.196	0.224
0.3	0.197	0.004	0.189	0.205	0.184	0.210
0.4	0.193	0.004	0.185	0.201	0.180	0.205
0.5	0.194	0.004	0.186	0.203	0.181	0.207
0.6	0.202	0.005	0.193	0.211	0.188	0.216
0.7	0.218	0.005	0.208	0.228	0.202	0.234
0.8	0.241	0.006	0.229	0.252	0.222	0.259
0.9	0.285	0.008	0.270	0.300	0.261	0.309
*Panel B. Characteristics*						
Mean Quantile	0.210	0.005	0.201	0.219		
0.1	0.246	0.005	0.236	0.256	0.234	0.258
0.2	0.212	0.004	0.204	0.221	0.201	0.223
0.3	0.192	0.004	0.184	0.201	0.182	0.203
0.4	0.181	0.004	0.173	0.189	0.171	0.191
0.5	0.177	0.004	0.169	0.185	0.166	0.187
0.6	0.177	0.004	0.169	0.185	0.166	0.188
0.7	0.185	0.004	0.176	0.193	0.173	0.196
0.8	0.199	0.005	0.190	0.209	0.187	0.211
0.9	0.237	0.006	0.225	0.250	0.221	0.253
*Panel C. Implicit prices*						
Mean Quantile	0.024	0.004	0.017	0.032		
0.1	$-$0.005	0.006	$-$0.017	0.007	$-$0.025	0.015
0.2	$-$0.003	0.004	$-$0.011	0.005	$-$0.016	0.011
0.3	0.004	0.003	$-$0.002	0.011	$-$0.007	0.016
0.4	0.011	0.003	0.005	0.017	0.001	0.022
0.5	0.018	0.003	0.012	0.023	0.008	0.027
0.6	0.025	0.003	0.019	0.031	0.015	0.035
0.7	0.033	0.003	0.027	0.040	0.023	0.044
0.8	0.041	0.004	0.034	0.049	0.029	0.054
0.9	0.048	0.005	0.039	0.057	0.032	0.064

**Fig. 4:**
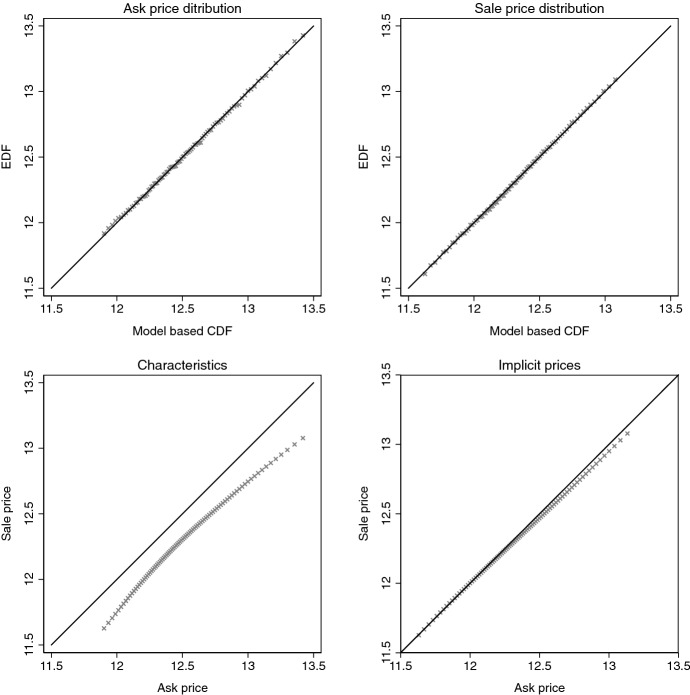
Q–Q plots for price distributions. Upper-left (right) panel compares ${\hat{F}}_{a|a}$ (${\hat{F}}_{s|s}$) to the EDF of the ask (sale) price, where ${\hat{F}}_{j|k}$ is estimated from Eq. (4). Lower-left (right) panel compares ${\hat{F}}_{a|a}$ (${\hat{F}}_{a|s}$) to ${\hat{F}}_{a|s}$ (${\hat{F}}_{s|s}$). Solid black line is the 45 degree line

Estimates of $\text{ E }[p_a] - \text{ E }[p_s]$ and $Q_a(\tau ) - Q_s(\tau )$, the left-hand sides of the mean and quantile differences to be decomposed, are reported in Panel A of Table 4. The estimated markup at the median is slightly lower than the markup reported in Fig. 1. This is because the markups are estimated from Eq. (4), rather than the empirical distribution functions (EDFs) of ask and sale prices. The upper-left (right) panel of Fig. 4 shows a Q–Q plot for the ask (sale) price distribution estimated from Eq. (4) and the EDF. For, both, ask and sale prices the distribution ${\hat{F}}_j(p)$ resembles closely the corresponding EDF. This is reflected in the estimated markups, which exhibit a similar U-shaped pattern as the markups in Fig. 1.

Panels B and C of Table 4 show the estimated contributions of characteristics and implicit prices to the corresponding differences of Panel A. As indicated by the Q-Q plots in the lower panel of Fig. 4, the contribution of characteristics differences in the two data sets accounts for the greater part of the markups. Nonetheless, according to the pointwise *and* uniform confidence bands, implicit price contribute also to the markups for $\tau >0.3$, at least, at the 0.05 level. At the means, pricing difference contribute a tenth to the markup. This corresponds to 2.4 percentage points, which is in the range of markups reported in papers that worked with matched ask and sale data (see Fn. 3). At the medians, the contribution is of similar magnitude. At lower quantiles, the contribution can be statistically zero, whereas it can be up to a fifth at higher quantiles.

The analysis shows that implicit prices play a significant role for the ask price markups. But the analysis also confirms that houses in the ask data are of better quality than those in the sale data. We can only speculate about reasons for this selectivity. A platform requires that households have access to the internet and use it for property search. Some households might rely on traditional search channels, such as newspapers or real estate agent registers. Selectivity could arise if the choice of channel is correlated with the house characteristics that households are interested in. Analysis of data from the German Socio-Economic Panel (GSOEP) for 2007–2015 shows that households in Berlin who use the internet spend on average more on housing, even after controlling for age, education income, and household size.^16^ It is therefore likely that platforms attract users that are prepared to pay for better houses. On the customer side, the majority (82.9%) of listings in the ask data are for existing houses. The rest are for houses that are either new or at most one year old; about three quarters of these houses are marketed directly by the developer. Existing houses are predominantly (87.9%) marketed by real estate agents. As agents will receive a closing fee in case of a successful match, they have an interest to chose the right marketing channel for a particular property. Compared to the few existing houses that are marketed without fee, indicating that no agent is involved, agents list houses that are on average larger, although also slightly older. As platforms are characterised by indirect network effects, these will enforce selectivity of the listed properties even more.^17^

In summary, the distribution of characteristics and the pricing of these characteristics are significantly different in the ask and the sale data. This makes it possible that research results are severely biased when ask data are used as a substitute for sale data.

## Hedonic regression applications

We examine the extent of the bias that is introduced when sale data are substituted with ask data for the three economic research areas: (1) estimating the willingness to pay for non-traded amenities, (2) automated valuation applications, (3) construction of price indexes. For each of the three applications, we compare the results we obtain from ask data with those we obtain from sale data. We explain the empirical methodology first, followed by the presentation of the empirical results.

### Methodology and implementation

#### The semiparametric hedonic model

Hedonic regression is the basis for each of the three economic applications. Fully parametric linear models can impose restrictions that do not accommodate the unknown data generating process. Such models impose also restrictive assumptions on preferences (Ekeland et al. 2004). Nonparametric models provide full flexibility, but can suffer from the curse of dimensionality. Semiparametric models place *some* structure on the functional form and are a good compromise, see, for example, Bontemps et al. (2008).^18^ Our semiparametric additive regression model is

6 $$\begin{aligned} p = \mathbf {z} \varvec{\gamma } + f_{1}(AGE) + f_{2}(FA) + f_{3}(PA) + f_4(LAT,LON) + \varepsilon \end{aligned}$$

For a given data set and observation, *p* is the price reported, the row vector $\mathbf {z}$ contains dummy variables for the constant, quarters, discrete house characteristics, and—depending on the specification—for the districts. The column vector $\varvec{\gamma }$ contains the coefficients for these discrete characteristics. The impact of the continuous characteristics on the price are considered by smooth, but unspecified, functions $f_j$. The error term $\varepsilon$ represents the part of the price left unexplained by the model. The continuous characteristics are building age ($AGE$), floor ($FA$) and plot ($PA$) area, longitude and latitude coordinates ($LAT,LON$). We add $f_5(NOI)$ to Eq. (6) once we examine the WTP for the local noise level (*NOI*).^19^

We model the nonparametric functions in Eq. (6) with penalised regression splines

7 $$\begin{aligned} f_{j}(x) = \sum _{k=1}^{K_j} b_{jk}(x) \beta _{jk} = \mathbf {b}_j(x) \varvec{\beta }_j \end{aligned}$$

where $\mathbf {b}_{j}(x)$ is the row vector of $K_j$ basis functions evaluated at *x* and $\varvec{\beta }_j$ is the column vector of coefficients (Wood 2017). The vector of coefficients determines the shape of $f_j$ and has to be estimated. We use cubic splines as basis for the univariate functions in Eq. (6) and a thin plate spline for the function of the geo-coordinates (in which case *x* is a vector). Given the basis dimensions $K_j$, the vector of all basis functions $\mathbf {b}(\mathbf {x})$ with $\mathbf {x}$ the vector of continuous characteristics of an observation, the stacked coefficient vectors $\varvec{\beta }$ and $\varvec{\gamma }$ are then estimated separately for each of the two data sets as

8 $$\begin{aligned} \left( \hat{\varvec{\gamma }},\hat{\varvec{\beta }}\right) = \arg \min _{\varvec{\gamma }, \varvec{\beta }}\left[ \sum _{n=1}^N \left\{ p_n -\mathbf {z}_n\varvec{\gamma }- \mathbf {b}(\mathbf {x}_n) \varvec{\beta }\right\} ^2 + \sum _{j=1}^J\lambda _j \varvec{\beta }_j'\mathbf {D}_j \varvec{\beta }_j\right] \end{aligned}$$

The term $\varvec{\beta }_j'\mathbf {D}_j\varvec{\beta }_j$ evaluates $\int \left[ f_j^{''}(x)\right] ^2 \hbox {d}x$ and becomes large if $f_j$ is very wiggly and small if the function is fairly straight.^20^ The smoothing parameter $\lambda _j$ determines the degree at which wiggliness of the estimate of $f_j$ is penalised. To prevent excess smoothing, we select the parameters with a double cross-validation criterion (DCV) (Wood 2017, pp. 260). The web-appendix (B) provides details on the estimation procedure.

#### Willingness to pay

Once the hedonic regression is estimated, we compute for each characteristic the average marginal willingness to pay (WTP) in monetary terms and compare by how much the estimates differ between the two data sets. For a continuous characteristic, we use

9 $$\begin{aligned} \text{ WTP}_j = \frac{1}{N} \sum _{n=1}^N \frac{{\partial \hat{f}}_j(x_{j,n})}{\partial x_j} \exp \left\{ {\hat{p}}(\mathbf {z}_n,\mathbf {x}_n)\right\} \end{aligned}$$

where we compute the derivative numerically with finite differences and ${\hat{p}}(\cdot )$ is the prediction from Eq. (6). For a discrete characteristic, we use

10 $$\begin{aligned} \text{ WTP}_j= \frac{1}{N} \sum _{n=1}^N \left( \exp \left\{ {\hat{\gamma }}_j\right\} -1\right) \exp \left\{ {\hat{p}}(\mathbf {z}_{n,j}^0,\mathbf {x}_n) \right\} \end{aligned}$$

where $\mathbf {z}_{n,j}^0$ is the discrete characteristic vector for observation *n* with the entry for *j* set to zero.^21^ To compute heteroscedasticity-robust standard errors for the WTP estimates, we use the pairs bootstrap (Freedman 1981).

#### Automated valuation

We use a rolling window design to split the data into estimation and validation samples. The first validation sample contains all sale observations from 2009Q1. To predict prices with the ask data, we use the observations from 2007Q2 to 2009Q1, estimate the pricing function ${\hat{p}}_a(\cdot )$ from Eq. (6), and assess this function at the characteristics $(\mathbf {z},\mathbf {x})$ of the observations in the first validation sample. The choice of estimation sample considers that ask data are available instantly. For the sale data, we proceed similarly, but use observations from 2007Q1 to 2008Q4 to estimate ${\hat{p}}_s(\cdot )$. The lag of one quarter considers that sale data are not instantly available. The validation and estimation windows are then rolled out quarterly and predictions are computed until the last validation sample in 2015Q4 is reached. The price predictions for the final sample are computed for the ask (sale) data set based on the estimated price function 2014Q1 to 2015Q4 (2013Q4 to 2015Q3). We compute the prediction errors $e_{j,n}\equiv p_{s,n}-{\hat{p}}_j(\mathbf {z}_n,\mathbf {x}_n)$ from ask ($j=a$) and sale ($j=s$) data for further analysis.^22^

#### Price index construction and nowcasts

We compute constant-quality price indices from the ask and sale data using the hedonic imputation method.^23^ We therefore use a rolling window design similar to the one in Sect. 3.1.3. The first estimation sample contains all ask (sale) observations from 2007Q1 to 2008Q4. Given the estimated price function ${\hat{p}}_j(\cdot )$ from Eq. (6), we impute the price of a house with reference characteristics $(\mathbf {z}_0,\mathbf {x}_0)$ for each quarter in the estimation sample. The estimation sample is then rolled forward one quarter and the fitted price function is used to impute the value of the reference house in 2009Q1. The rolling imputations are continued until the last quarter 2015Q4 is reached. The ask (sale) price index is then computed for a house with fixed characteristics and covers the period 2007Q1 to 2015Q4.

As the sale price index become available only with the delay, whereas the ask price index becomes available in real time, we examine the potential for nowcasting with the regression

11 $$\begin{aligned} \varDelta I^s_t =\phi _0+\phi _1 \varDelta I^a_t +\phi _2 \varDelta I^a_{t-1} + \phi _3 \varDelta I^s_{t-1} +\epsilon _t \end{aligned}$$

where $I^a_t$ ($I_{t}^s$) is the ask (sale) price index for period *t* and the operator $\varDelta$ produces either the quarter-on-quarter or the year-on-year growth rate. We estimate Eq. (11) with OLS and use robust standard errors to control for further structure in the short series. Inclusion of $\varDelta I^s_{t-1}$ allows to examine whether the ask price index can improve upon an univariate forecast of the sale price index. As the index series have only 36 observations each, the examination will be limited.

### Empirical results

#### Willingness to pay

We examine whether WTP estimates from the ask and the sale data differ statistically and economically. It is known that estimates from hedonic regressions can suffer from omitted variable bias, but Kuminoff et al. (2010) have shown in a simulation study that spatial modelling can reduce such bias.^24^ We consider two spatial models in our regressions. First, as observations in the ask data may provide only coarse location information, we run regressions that model the spatial structure with district dummies as spatial fixed effects. Second, we run regressions that model location finely with the geospatial function $f_4(LAT, LON)$.^25^ As some observations report no coordinates, these regressions are fitted with smaller samples.^26^

**Fig. 5:**
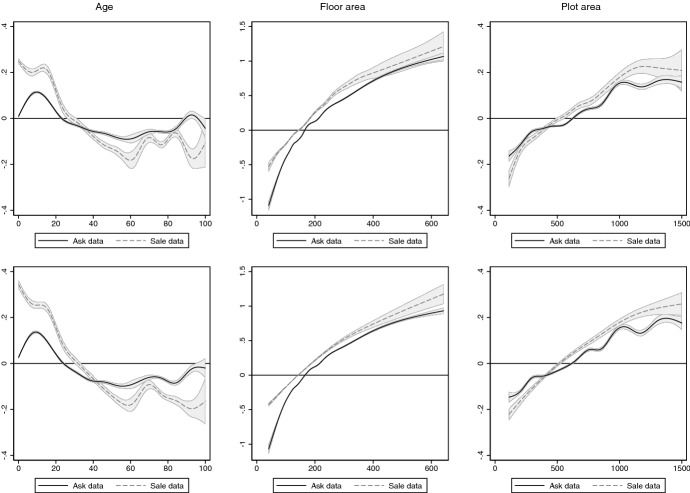
Estimates of components of semiparametric regression model. Upper panel shows estimates of $f_1(AGE)$, $f_2(FA)$, and $f_3(PA)$ for regression in Eq. (6) that uses spatial fixed effects. Lower panel shows estimates for the same functions, but controls with the geospatial function $f_4(LAT,LON)$. The corresponding estimated functions are shown in Fig. 6. Noise variable *NOI* is not included in the regressions. Functions are normalised to have a mean of zero. Shaded areas are pointwise confidence intervals at the 0.95 level, computed using heteroscedasticity robust standard errors

Figure 5 shows the estimates of the functions $f_j$ in Eq. (6) for the three continuous house characteristics age, floor and plot area. The upper (lower) panel shows the estimates that result when location is modelled with spatial fixed effects (geospatial function).

**Fig. 6:**
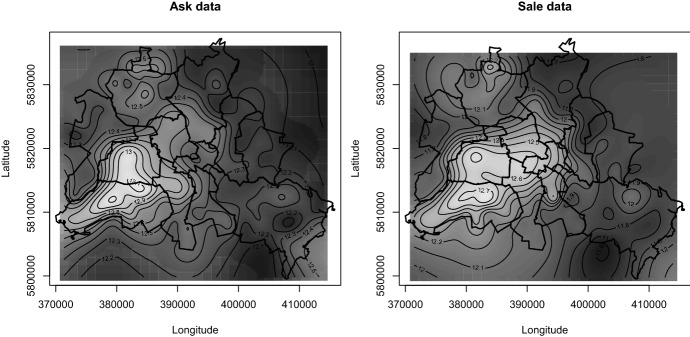
Location value surface. Shows contour plot of estimated geospatial functions ${\widehat{f}}_4(LAT,LON)$ from ask (left panel) and sale data (right panel). Location value surface is evaluated at median values of continuous house characteristics and modal values of discrete house characteristics

Evidently, as the ask data have more observations, the functions are estimated more precisely. It also seems that all functions become smoother once the geospatial function is used. The functions for the areas, while not identical, seem similar whether estimated with ask or sale data. However, we expect these functions to increase monotonically, but the function for plot area estimated with ask data shows several ups and downs that counter intuition. The functions for age differ substantially. When estimated with sale data, the function falls monotonically up to an age of 60 years, where it increases and falls again. This non-monotonic shift can be explained by a premium for houses that survived WWII. When estimated with ask data, the function increases over the first ten years, which is counterintuitive, and exhibits for higher ages several ups and downs. This erratic behaviour is hard to explain other than being an artefact of the ask data. Figure 6 shows contour plots of the estimated geospatial functions. Both look similar and pick up the high quality of amenities in the south-westerly neighbourhoods of Berlin. Assessed at the locations of the sold houses, the correlation between the two estimated functions is high ($\rho =0.94$).

**Table 5:** Willingness to pay for house characteristics. Reports WTP estimates and regression diagnostics for penalised least squares estimates of Eq. (6). WTPs for continuous (discrete) house characteristics are computed with Eq. 9 (Eq. 10). Standard errors are computed using the pairs bootstrap with 200 replications. $${\bar{R}}^2$$ is the adjusted coefficient of determination. *DCV* is the double cross-validation score. Significant at $$^{***}$$0.001 level, $$^{**}$$0.01 level, $$^{*}$$0.05 level

	Ask data				Sale data					
	(1)		(2)		(3)		(4)		(5)	
	WTP	SE	WTP	SE	WTP	SE	WTP	SE	WTP	SE
Age	243.99$$^{***}$$	47.25	$$-$$78.96	43.88	$$-$$1173.73$$^{***}$$	103.73	$$-$$1461.39$$^{***}$$	106.05	$$-$$1562.89 $$^{***}$$	110.14
Floor area	1419.07$$^{***}$$	10.39	1293.92$$^{***}$$	7.41	1047.99$$^{***}$$	17.46	906.73$$^{***}$$	14.82	830.24$$^{***}$$	16.87
Plot area	1465.18$$^{***}$$	7.95	1380.25$$^{***}$$	7.46	1152.82$$^{***}$$	18.14	1011.70$$^{***}$$	14.60	940.84$$^{***}$$	16.79
Detached	6881.76$$^{***}$$	1424.74	17771.60$$^{***}$$	1263.12	$$-$$7918.00$$^{**}$$	2468.32	4130.49	2198.43	6546.97$$^{**}$$	2999.16
Semi-detached	$$-$$1042.73	1205.12	5074.97$$^{***}$$	1011.21	$$-$$8160.82$$^{**}$$	2630.25	3741.82	2315.45	3705.94	2158.45
Listed									12599.23$$^{**}$$	3702.38
Prefabricated									$$-$$6544.52$$^{***}$$	1686.19
Converted attic									3426.77$$^{**}$$	1141.96
Swimming pool									14744.26$$^{**}$$	5509.92
Flat roof									$$-$$5158.22$$^{***}$$	1426.43
No basement									$$-$$21888.12$$^{***}$$	1724.46
Backland develop.									396.67	1363.94
Waterfront									59947.35 $$^{***}$$	6417.56
Poor condition									$$-$$53313.91$$^{***}$$	2341.98
Good condition									29031.33$$^{***}$$	1657.96
Poor amenities									$$-$$6263.71$$^{***}$$	1720.23
Good amenities									16298.74$$^{***}$$	2243.77
Excel. amenities									37654.98$$^{***}$$	9613.84
$f_4(LAT,LON)$		No		Yes		No		Yes		Yes
District dummies		Yes		No		Yes		No		No
Year dummies		Yes		Yes		Yes		Yes		Yes
Buyer/Seller dummies		No		No		No		No		Yes
*DCV*		0.048		0.039		0.068		0.056		0.050
${\bar{R}}^2$		0.775		0.816		0.665		0.731		0.761
*N*		68,070		59,502		12,524		12,218		12,218

Table 5 presents in columns (1)–(4) the estimated WTPs for the core house characteristics. We note that the standard errors for the WTPs are smaller when the ask data are used (result of the larger sample sizes). In case of the ask data, only the use of the geospatial function leads to an intuitive negative WTP for age, although it remains insignificant at the usual levels (2). The counterintuitive age function from Fig. 5 shows up here. When estimated with the sale data, the estimated WTP for age is both times negative at the usual significance levels, irrespective of the spatial modelling approach. Regarding the house types, terraced is the reference type and the WTPs for the other two types aligns with intuition only when the geospatial function is included in the regressions. As to be expected from Kuminoff et al. (2010), it seems that the geospatial function deals with omitted variable bias, as it leads to more intuitive WTP estimates.

In the examination so far, we have used only those house characteristics that are in the ask as well as in the sale data. The sale data are, however, of better quality and contains also information on other characteristics and on parties involved in a transaction, see the second part of Panel A in Table 2. (5) in Table 5 gives the WTP estimates when we no longer ignore this information. The WTP estimates for the formerly omitted variables seem sensible. The regression continues to use the geospatial function to control for other omitted variables. (5) is our most complete specification and we use its estimates as benchmark. Comparison of (4) with the benchmark shows that the formally omitted characteristics from the sale data have only a fairly small effect on the estimated WTPs for the core characteristics. The point estimates deviate by no more than 10% from the benchmark. Things look different when we compare (2) with the benchmark. In all but one case, the estimates from the ask data are between 1.4 and 2.7 times the benchmark. Inflated WTP estimates can be expected given the ask data’s dominant characteristics and implicit price distributions. The only exception is the WTP for age, which is only 0.1 times the benchmark. This reflects the counterintuitive age function that results for the ask data.

**Fig. 7:**
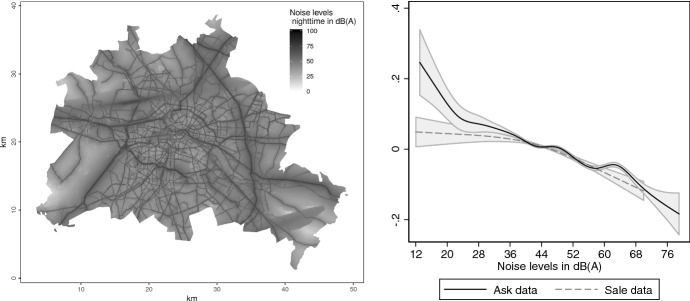
Noise levels and WTP. Left panel shows noise levels in decibel (dB(A)) in Berlin at night for 2012. The data comes from Berlin’s Senate Department for Urban Development and Housing. Right panel shows estimates of the function $f_5$ from specifications (2) and (5) in Table 5, when including $f_5$. Shaded areas are 0.95 pointwise confidence intervals, computed using heteroscedasticity robust standard errors

Finally, we examine what such deviations imply for benefit assessment. Figure 7 plots nightly noise levels in Berlin for 2012, the darker the shading, the higher the noise. For example, the dark strip from left to right in the upper part corresponds to the noise emitted by Otto Lilienthal airport in Tegel; the noise emitted by inner-city motorways is also visible.

Estimated with sale data, the function $f_5(NOI)$ in Fig. 7 stays reasonably flat at zero up to a level of 50db—the level of noise in a quiet suburban neighbourhood—and becomes increasingly negative at higher noise levels. Estimated with ask data, however, the function puts a doubtful premium on silence—30db corresponds to rustling leaves—and exhibits non-monotonic behaviour. The estimated WTPs that result from these two functions are reported in Table 6.

**Table 6:** Willingness to pay for noise levels. Reports WTP estimates and regression diagnostics for penalised least squares estimates of Eq. (6). WTPs are computed with Eq. (9). Specification for ask (sale) data identical to (2) ((5)) from Table 5 plus noise function $$f_5(NOI)$$. Standard errors are computed using the pairs bootstrap. Number of bootstrap replications is 200. $${\bar{R}}^2$$ is the adjusted coefficient of determination. *DCV* is the double cross-validation score. Significant at $$^{***}$$0.001 level, $$^{**}$$0.01 level, $$^{*}$$0.05 level

	Ask data		Sale data	
	WTP	SE	WTP	SE
Noise level	$$-1141.27^{***}$$	79.73	$$-846.05^{***}$$	115.57
*DCV*		0.039		0.049
${\bar{R}}^2$		0.819		0.763
*N*		59,502		12,218

The estimates are negative—noise is a disamenity—and significantly different (Welch’s t-test has a *p*-value of 0.000).^27^ The difference between the point estimates seems economically small, which ignores that noise usually affects many households. The difference becomes EUR532,900 per $\text{ km}^2$ after we factor in that in Berlin the average density is 1700 households per $\text{ km}^2$. Obviously, a policy maker who uses the cost-benefit criterion to decide on a night flight ban may come to the wrong decision when the benefit is estimated with ask data.

#### Automated valuation

Table 7 presents performance measures for the out-of-sample predictions for regressions fitted separately to ask and sale data.

**Table 7:** Assessment of prediction errors. Shows performance statistics for 9,152 out-of-sample prediction errors. $\pm 10\%$ ($\pm 25\%$) reports the proportion of errors which are in absolute terms no larger than 10% (25%)

Data	MSE	Bias	Var.	Med.	MAE	$\pm 10\%$	$\pm 25\%$
Ask	0.077	$-$0.035	0.076	$-$0.021	0.214	0.304	0.670
Sale	0.051	0.007	0.051	0.017	0.174	0.368	0.752

**Fig. 8:**
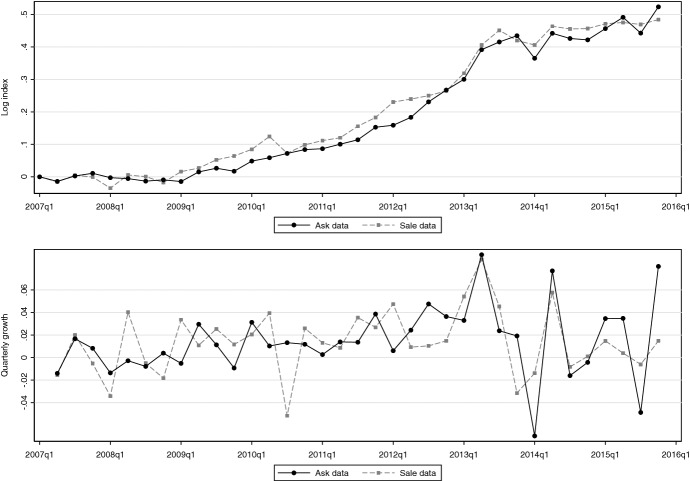
Quarterly quality-adjusted house price indices. Upper panel shows ask and sale price indices for Berlin 2007Q1–2015Q4, lower panel shows quarter-on-quarter growth rates. The growth rate of the ask (sale) price index is 1.5% (1.4%) with volatility of 3.2% (2.8%)

The prediction errors $e_{a,n}$ do not perform as well as the errors $e_{s,n}$. The threshold proportions are less than 0.9 times of those for the latter and the MSE is 1.5 times as large. The negative bias of the errors $e_{a,n}$ is not surprising given that the distribution of implicit prices in the ask dominates those in the sale data.^28^ However, the errors $e_{s,n}$ show also bias, which reflects the quarterly lag of the data used for estimation. The magnitude of the bias is half of the quarter-on-quarter growth rate of quality-controlled sale prices, see Fig. 8. One could suspect that the differential performance of the errors comes mainly from the tendency of ask prices to be larger than sale prices. However, the bias is fairly unimportant for the MSE of the two sets of errors. The inferior performance of the $e_{a,n}$ errors comes mainly from their high variance, the result of fewer variables that can be used and their tilted pricing differences.

#### Price indices and nowcasts

Figure 8 shows the quality-controlled ask and sale price indices, based, respectively, on specifications (2) and (5) from Table 5. The two indices have overall the same upward trend, but the trend masks some differences that are visible in the quarter-on-quarter growth rates. As both indices control for observed characteristics, these differences are due to differential valuations, wider coverage of characteristics and a random element.

Table 8 assesses the strength of the relation between the two indices and gives results for the price index growth rate regression from Eq. (11). Panel A (B) reports results for quarter-on-quarter (year-on-year) growth rates. As (1) shows, the contemporaneous rates of the two indices are positively correlated, but the relation is stronger for the year-on-year than the quarter-on-quarter growth rates (${\hat{\rho }}=0.80$ versus ${\hat{\rho }}=0.51$). The former are usually less volatile, which makes it more likely to detect a relationship—if it exists—in small samples like ours. Interestingly, the coefficient on $\varDelta I_{t}^a$ is significantly less than one, across (1)–(5) in, both, Panel A and B. This shows that the ask price index is not a perfect substitute for the sale price index.^29^ For the quarter-on-quarter growth rates, adding the lagged growth rates of the ask *or* sale price does not improve explanatory power, see ${\bar{R}}^2$ in (2)–(4). In (5), however, the coefficients on $\varDelta I_{t-1}^a$ *and* $\varDelta I_{t-1}^s$ are statistically significant at the 0.05 level, and $\varDelta {\hat{I}}^s_t=0.006+0.569\varDelta I_t^a +0.324\varDelta I_{t-1}^a-0.314\varDelta I_{t-1}^s$ produces the best in-sample fit. For the year-on-year growth rates, the lag of the sale price has on its own already high predictive power, see (3). But even in this instance, the inclusion of the current growth rate of the ask price index improves explanatory power, see ${\bar{R}}^2$ in (4) and (5). For a nowcast, it is best to use $\varDelta {\hat{I}}^s_t=0.014+0.531\varDelta I_t^a +0.268\varDelta I_{t-1}^s$.

**Table 8:** Nowcast regressions. Reports estimates of Eq. (11). Asymptotic *p*-value is for the two-sided null hypothesis that the respective coefficient is zero. *t*-statistics are computed using Newey–West standard errors, at most four lags. ${\bar{R}}^2$ is the adjusted coefficient of determination

	(1)		(2)		(3)		(4)		(5)	
	Coeff.	*p*-val.	Coeff.	*p*-val.	Coeff.	*p*-val.	Coeff.	*p*-val.	Coeff.	*p*-val.
	*Panel A. Quarter-on-quarter*									
$\varDelta I^a_t$	0.445	0.001	0.464	0.000			0.448	0.002	0.569	0.000
$\varDelta I^a_{t-1}$			0.136	0.264					0.324	0.023
$\varDelta I^s_{t-1}$					0.013	0.945	$-$0.096	0.490	$-$0.314	0.020
Constant	0.007	0.011	0.006	0.066	0.015	0.000	0.009	0.002	0.006	0.084
${\bar{R}}^2$		0.241		0.222		$-$0.031		0.210		0.260
*N*		35		34		34		34		34
	*Panel B. Year-on-year*									
$\varDelta I^a_t$	0.734	0.000	0.519	0.000			0.531	0.000	0.493	0.000
$\varDelta I^a_{t-1}$			0.238	0.011					0.102	0.332
$\varDelta I^s_{t-1}$					0.664	0.000	0.268	0.010	0.205	0.124
Constant	0.016	0.033	0.017	0.032	0.023	0.000	0.014	0.061	0.015	0.067
${\bar{R}}^2$		0.633		0.642		0.469		0.654		0.645
*N*		32		31		31		31		31

Taken together, the examination provides evidence that an ask price index is not a substitute for a sale price index but can lead to better nowcast. Longer time series are needed to obtain clearer in-sample results and to extend the examination to out-of-sample nowcasts.

#### Robustness checks

We conducted several robustness checks to assess the sensitivity of our results to methodological choices, see the web-appendix (D) for details. First, we examined the sensitivity of the markup decomposition to a different specification of the quantile regressions. A linear model does not alter the results much and gives an even stronger role to the implicit prices for the markups. This implies that our reported results from the polynomial model are conservative. Second, we estimated the WTP for non-overlapping sub-periods of our sample. We found little difference between the WTP computed with individual WTP estimates for sub-samples and estimates for the full sample. The former estimates have higher standard errors, however, which makes them inferior to the ones reported here. We also found statistical evidence that the mark-ups of WTP estimates from ask data over estimates from sale data remains the same over the sub-samples. Third, instead of using a constant factor to convert the floor area from the interior area, we used an estimated conversion function that considers building age and type. We estimated this function with transaction data that have information on both area characteristics. The floor areas imputed with the estimated function are highly correlated with those that use the constant conversion factor. Fourth, the estimates in Fig. 5 might suggest that commonly applied parametric specifications, such as polynomials, could be appropriate. However, we found that such a specification leads inferior predictive performance compared to the semiparametric model we use here.

## Conclusion

Unlike transaction data, listings data are readily available from platforms. This makes it an appealing new source of data for housing market research. Researchers have already begun to use ask data in the three established applications of WTP estimation, automated valuation, and price index construction. This paper examined whether it is valid to do so.

The literature on search and bargaining in housing markets suggests that ask data will differ in important aspects from sale data. Our empirical investigation produced three important insights into this difference. First, distributions of ask and sale prices differ, mainly because the composition of characteristics differs between the data sets. But the estimates of implicit prices also differ between the two data sets. This indicates that ask data might not be a valid substitute for sale data in the three applications. Indeed, WTP estimates for house characteristics and local noise levels from the two data sets can be considerably different. Second, ask data are not very useful to predict market values of individual houses, as the predictions suffer from upward bias and large error variance. Third, we find that an ask price index is not a substitute for a sale price index. It is, however, a complement to the latter, as we obtain evidence that it is useful for nowcasting. No doubt, listings data can be useful for research, but ask data are no replacement for sale data, at least in the three applications that we have considered in this paper.

There are potential avenues for future research. We used ask data from only one platform, albeit the largest. Analysis of data from other platforms could provide further insights into the selection bias of characteristics that we observed. It would not deal with the problem that some sellers simply do not advertise on platforms. Recent events might change this in the near future. In 2015, the German regulator permitted the merger between the second and the third largest platform (see Fn. 5), which should increase competition for such sellers. With the onset of the Coronavirus pandemic in March 2020, several platforms allowed private customers to list at no charge. While not permanent, this measure could bring new customers who stay even once they are charged. This could make ask data more representative. However, the selection bias is not responsible for the result that the estimated implicit prices differ between the two data sets. Genesove and Mayer (2001) and Bigelow et al. (2020) provide explanations for this result. Sellers (and agents) can misperceive the value to others of characteristics that are salient to them (the price they paid, the importance of amenities). They will learn the value to others during the bargaining process. The element of bargaining is—yet—missing from platforms. Still, as platforms provide a wide range of visual and textual information of comparable houses on the market, sellers (and agents) might become able to distinguish better how the average seller values characteristics that they hold dear. In this case, we expect that the difference in implicit prices should shrink if platforms make the market more transparent.

## Supplementary Information

Below is the link to the electronic supplementary material.

Supplementary material 1 (pdf 629 KB)

## Footnotes

1 IS24 is the largest platform in Germany, followed by Immowelt. Both are also active in Austria and Switzerland. Platforms in other countries are Zillow (USA) and Rightmove (UK).

2 It is not easy to detect this. For instance, press releases of IS24 (23.04.2020) and Immowelt (Nürnberg 23.06.2020) use the terms sale price (*Kaufpreis*) and ask price (*Angebotspreis*) interchangeably.

3 In the data of Shimizu et al. (2016), the average ask price is about 25% (36%) higher than the average registered sale price for successful (unsuccessful) listings. In the matched data of Haurin et al. (2010) and Carrillo (2012), the average ask price is 4% and 2% higher than the average sale price, respectively. This relationship between ask and sale price will hold for the majority of transactions, but the share might vary with the market. Han and Strange (2014, Table 2), for instance, find a share of 91% during the US market bust (2007–2010) compared to 86% during the boom (2003–2006).

4 Admittedly, seller and buyer characteristics are rarely reported in transaction data sets. But this does not invalidate the argument. Our transaction data contains some information on sellers and buyers.

5 Bundeskartellamt, Fallbericht B6-39/15 (25.06.2015). In 2017, IS24 listed 470,000 properties and had about 13m visitors per month, 1.9 and 1.6 times as many as the next largest competitor (Scout24 2017).

6 Possible terms are: two weeks, one month, three months. Listings can also be *premium* or *basic*. Premium listings permit a detailed presentation of the property and the ad will be placed more prominently on the web page.

7 The GAA data reports for most observations *exclusively* the exterior floor area; the IS24 data reports *exclusively* the interior area. The GAA suggests a factor of 1.25 to convert interior area to floor area (Gutachterausschuss für Grundstückswerte 2011, p. 44). We apply this factor to the IS24 observations, but examine an alternative in the robustness checks.

8 The bounds are differentiated further by location, house type, and vintage of the building. We collate the bounds from annual reports published by the GAA, see the web-appendix (A).

9 The mark-up at quantile $\tau$ is $\exp \{p_a(\tau )-p_s(\tau )\}-1\doteq p_a(\tau )-p_s(\tau )$; we estimate the right-hand side with quantile regressions of prices on a constant and an indicator that is one (zero) for the ask (sale) price.

10 Stochastic dominance means $\text{ Prob}_a\{p_a\geqslant p\}\geqslant \text{ Prob}_s\{p_s\geqslant p\}$. This is equivalent to $1-F_a(p)\geqslant 1-F_s(p)$, which gives the inequality in the text.

11 The age variable is discrete and the KS results could be too conservative. We conduct also Wilcoxon–Mann–Whitney tests, which lead to the same individual and joint test outcomes as those from Table 3.

12 t-tests (not reported) show that the proportions of the three house types are different between the data at the usual significance levels.

13 Shimizu et al. (2016) plot point estimates for Eq. (3) and test whether differences between price and valuation distributions of ask and sale data are zero (the latter test ignores that hedonic coefficients are estimated). They do not test for stochastic dominance, although their Fig. 6 indicates that it might exist for ask over sale prices.

14 The quantiles in Eq. (4) follow $\tau _g=c+(g-1)(1-2c)/(G-1)$. We set $c=0.01$ and $G=200$. Trimming at *c* avoids estimation of tail quantiles (Koenker 2005, p. 148).

15 At the means, we use the Blinder–Oaxaca decomposition. We estimate the implicit prices by running a linear regression of the price on the continuous core variables and dummies for the house type, district, and year. We include the continuous variables as third-degree polynomials, which is analogous to the quantile regressions used to estimate Eq. (4).

16 Per year, the GSOEP covers about six hundred households from Berlin. We run a (unreported) regression of housing consumption on a binary indicator for internet access—as a proxy for use—and controls for the household head’s age and education, household income and size, and year effects. The coefficient on internet access is positive and statistically significant at the 0.01 level.

17 This helps to understand the selectivity of the IS24 data, but does not explain why *this* platform attracts listings of houses of better quality. A conversation with a representative of an agent trade body pointed to the role of different pricing strategies of competing platforms. This is an area for further research.

18 Haupt et al. (2010) find that a log-log specification performs better out-of-sample than semi- and nonparametric specifications (Anglin and Gencay 1996; Parmeter et al. 2007). The house transactions used in these studies contain only one continuous characteristic. As we work with up to six continuous characteristics, we expect that parametric restrictions will have a detrimental effect on performance. Our robustness analysis points in this direction.

19 The regression model of Eq. (6) has been referred to as a local regression model in the real estate literature (Clapp 2003, 2004) or a geoadditive model in the geostatistical literature (Kammann and Wand 2003). We refer to it as a semiparametric additive model, the name commonly used in the statistics literature. Other modelling approaches to control for spatial effects include geographically (or locally) weighted regression and spatial error or spatial lag models. See, for example, Sunding and Swoboda (2010) and Small and Steinmetz (2012) for applications to hedonic pricing and Hill and Scholz (2018) for price index construction.

20 Depending on the specification, *J* is either 4 or 5. The elements of $\mathbf {D}_j$ are discussed in Wood (2017, Sec. 5.3 and 5.5).

21 Note that each of the individual willingness to pay terms over which we sum on the right-hand sides of Eqs. (9) and (10) depends on *all* characteristics, including a property’s location. This implies that these terms will vary spatially if location matters.

22 Due to the estimation lag, ${\hat{p}}_s(\cdot )$ ignores the time dummy for the current quarter in $\mathbf {z}$.

23 See Hill (2013) for a recent survey on hedonic price indices and the web-appendix (C).

24 While omitted variable bias might pose problems for ask and sale data, it is not the source of the *comparative* differences in the application results.

25 Hill and Scholz (2018) find that finely graded postcode spatial fixed effects can work as well as a nonparametric function of coordinates, at least in a price index application.

26 13% (2%) of the ask (sale) observations have no coordinates, see bottom row of Table 5.

27 The point estimates correspond to a reduction of the average ask (sale) price by 0.4% (0.3%). Winke (2017, p. 1284) finds a reduction of 1.7% and reports that previous studies found reductions between 0.1% to 3.6%.

28 ${\hat{p}}_a(\mathbf {z},\mathbf {x})$ are effectively imputed ask prices and the bias of 3.5% falls well within the range of markups observed in studies that use matched data, see Fn. 3.

29 In (1), a test of the joint hypothesis $H_0: \phi _0=0 \text{ and } \phi _1=1$ is rejected at the 0.001 (0.01) level for quarter-on-quarter (year-on-year) growth rates. Backward-looking ask prices, as evidenced in Genesove and Mayer (2001), may contribute to the less than perfect relationship between the contemporaneous growth rates.
