# Supplementary material for: Real estate listings and their usefulness for hedonic regressions
Source: Empir Econ. 2021 Jan 13;61(6):3239–69. doi: 10.1007/s00181-020-01992-3 (PMC7805573; doi:10.1007/s00181-020-01992-3)
Supplement: Supplementary file 1 — Supplementary material 1 (pdf 629 KB) [file 181_2020_1992_MOESM1_ESM.pdf]

Web-appendix for 'Real estate listings and their usefulness for hedonic regressions'

INSERT JOURNAL and DOI

Jens Kolbe, Rainer Schulz, Martin Wersing,

and Axel Werwatz*

November 4, 2020

*Kolbe and Werwatz: Institut für Volkswirtschaftslehre und Wirtschaftsrecht, Technische Universität Berlin, Straße des 17. Juni 135, 10623 Berlin, Germany; jkolbe@tu-berlin.de and axel.werwatz@tu-berlin.de. Schulz and Wersing: University of Aberdeen Business School, Edward Wright Building, Dunbar Street, Aberdeen AB24 3QY, United Kingdom; r.schulz@abdn.ac.uk and martin.wersing@abdn.ac.uk.

Contents

A Bounds for characteristics **3**

B Semiparametric additive model **3**

C Hedonic price index **6**

D Robustness checks **7**

E Software packages **11**

A Bounds for characteristics

The annual reports of the GAA give information on the minimum, average, and maximum of the following continuous characteristics: floor area, plot area, and ratio of price to floor area; Gutachterausschuss für Grundstückswerte (2011, 2012, 2013, 2014, 2015). The information is based on houses transacted during the year previous to the respective publication date. The information is provided separately by house type (detached, semi-detached, terraced houses), detailed further by the part of the city the house is located in (East, West), and the vintage of the building (constructed before 1949 or since 1949). We use the information for the years 2010-2014 and choose the bounds for each of the three characteristics as the minimum of the minima and the maximum of the maxima separately for each house type, location, and vintage. Table A1 shows the resulting bounds that we place on the three continuous characteristics.

[Table A1 about here.]

B Semiparametric additive model

The nonparametric functions $f_j(x)$ for $j = 1, 2, 3, 5$ are modelled with the *cubic spline basis*

$$\begin{aligned} f_j(x) &= x\beta_{j1} + \sum_{k=2}^{K_j} |x - x_{jk}|^3 \beta_{jk} \\ &= \sum_{k=1}^{K_j} b_{jk}(x)\beta_{jk} = \mathbf{b}_j(x)\boldsymbol{\beta}_j \end{aligned} \tag{B1}$$

The $K_j - 1$ knots $x_{j2}, \ldots, x_{jK_j}$ are placed at the $K_j - 1$ equally spaced quantiles of x. There is no constant term, as it is considered in the vector $\mathbf{z}$. The two

natural spline constraints $\sum_{k=2}^{K_j} \beta_{jk} = 0$ and $\sum_{k=2}^{K_j} \beta_{jk} x_{jk} = 0$ are imposed, so that the second derivative of $f_j(\cdot)$ is zero outside $[x_{j2}, x_{jK_j}]$, which reduces the risk of extrapolation (Wood and Augustin 2002, p. 160). The nonparametric function $f_4(\mathbf{x})$ is modelled with—again without a constant term—the *thin plate spline basis*

$$\begin{aligned} f_4\left(\mathbf{x}\right) &= x_1\beta_{41} + x_2\beta_{42} + \sum_{k=3}^{K_4} b_{4k}(||\mathbf{x} - \mathbf{x}_{4k}||)\beta_{4k} \\ &= \sum_{k=1}^{K_4} b_{4k}(\mathbf{x})\beta_{4k} = \mathbf{b}_4(\mathbf{x})\boldsymbol{\beta}_4 \end{aligned} \tag{B2}$$

The two dimensional vector $\mathbf{x} = (x_1, x_2)$ contains the location coordinates and we use the functions $b_1(\mathbf{x}) = x_1$ and $b_2(\mathbf{x}) = x_2$ for compact notation. We use further $||\mathbf{u}|| = \sqrt{\mathbf{u}'\mathbf{u}}$ and

$$b_{4k}(||\mathbf{u}||) = \frac{1}{8\pi}||\mathbf{u}||^2 \log ||\mathbf{u}||$$

if $\mathbf{u}$ is two-dimensional as it is in our application (Wood and Augustin 2002, p. 171). The $K_4 - 2$ location knots $\mathbf{x}_{4k}$ are a subset of the actual locations of the observations. We explain the selection of this subset below.

Using the vector representations of the univariate and bivariate spline functions in the last lines of equations Eq. B1 and Eq. B2, we write our semiparametric model compactly as $p = \mathbf{z}\boldsymbol{\gamma} + \mathbf{b}(\mathbf{x})\boldsymbol{\beta} + \varepsilon$. *Given* K_j and λ_j for all j, the stacked $\boldsymbol{\beta}$ and $\boldsymbol{\gamma}$ coefficient vectors are estimated separately for each of the two data sets by penalized least squares

$$\left(\hat{\boldsymbol{\gamma}}, \hat{\boldsymbol{\beta}}\right) = \arg\min_{\boldsymbol{\gamma},\boldsymbol{\beta}} \left[\sum_{n=1}^{N} \{p_n - \mathbf{z}_n\boldsymbol{\gamma} - \mathbf{b}(\mathbf{x}_n)\boldsymbol{\beta}\}^2 + \sum_{j=1}^{J} \lambda_j \boldsymbol{\beta}_j' \mathbf{D}_j \boldsymbol{\beta}_j\right] \tag{B3}$$

Choice of knots. For the cubic spline basis, we set $K_j = 15$ for $j = 1, 2, 3, 5$. The 14 knots x_{jk} are set at the equally distanced quantiles of the respective

variable. This ensures that the variable range is adequately covered. For the choice of knots for the thin plate spline basis, we follow Wood (2003). First, for each of the two data sets, we draw a random sample with 2000 elements from the locations $\mathbf{x}_n$ of the N observations. Random sampling ensures that areas that have observations will be covered adequately. The randomly drawn locations are the initial set of knots $\mathbf{x}_{4k}$. We compute for each observation and initial knot $b_{4k}(\mathbf{x})$ and arrange them all in a matrix of dimension $N \times 2000$. Since the dimension of this matrix is too large to be computationally feasible, we use the eigenvalue decomposition described in Wood (2003) to reduce it. This results in $K_4 = 152$ knots for the geospatial regression spline.

Smoothing parameter. The smoothing parameter λ_j determines the degree at which wiggliness of the estimate of f_j is penalised. We select $\boldsymbol{\lambda} = (\lambda_1, \ldots, \lambda_5)$ by minimizing the double cross-validation (DCV) score

$$\hat{\boldsymbol{\lambda}} = \arg\min_{\boldsymbol{\lambda}} \frac{N \sum_{i=1}^{N} (p_i - \hat{p}_i(\boldsymbol{\lambda}))^2}{\{N - 1.5\mathrm{tr}\,(\mathbf{H}(\boldsymbol{\lambda}))\}^2} \tag{B4}$$

where $\hat{p}_i(\boldsymbol{\lambda})$ is the predicted price for a given set of $\boldsymbol{\lambda}$ values and $\boldsymbol{H} = ([\mathbf{ZB}]'[\mathbf{ZB}] + \mathbf{S}_{\boldsymbol{\lambda}})^{-1} [\mathbf{ZB}]'$ is the hat matrix of the penalized least squares estimator in Eq. B3. Here, $[\mathbf{ZB}]$ is the design matrix collecting the dummy variables and basis functions for the continuous variables. The matrix $\mathbf{S}_{\boldsymbol{\lambda}}$ collects the penalty terms (Wood 2017, pp. 249-50). DCV is a consistent estimator of the mean squared prediction error of the regression model and minimizing DCV prevents excess smoothing (Wood 2017, pp. 260-61).

Residual diagnostics. To assess whether our choice of the number of knots and their locations is sufficient to provide adequate flexibility, we fit cubic regression splines to the estimated residuals. To detect any remaining structure

that could be explained by expanding the number of knots in the original regression, we set the number of knots in these diagnostic regressions to $K = 29$, see Wood (2017, p. 343). Figures B1 and B2 show the ask and, respectively, sale price residuals fitted to the predicted price, age, floor and plot area.

[Figure B1 about here.]

[Figure B2 about here.]

For both sets of residuals, the fitted curves are (almost always) straight horizontal lines. The bumps in the smoothed ask price residuals can be explained by IS24 customers who tend to report rounded values for house characteristics; see also the kernel density estimates in Figure 2 in the main paper. This artefact of the ask data is still visible after setting the number of knots to $K = 43$ in the ask price regression (not reported). Moreover, the estimated functions $\hat{f}_j(\cdot)$, again using $K = 43$, are comparable to those in Figure 5 in the main paper (not reported). This is evidence that our choice of the number of knots is sufficiently large.

C Hedonic price index

We compute the quarterly price index as

$$I_t = \hat{p}_t(\mathbf{x}_0) - \hat{p}_0(\mathbf{x}_0) \tag{C1}$$

where $\hat{p}_t(\mathbf{x}_0)$ is the imputed log price for a house with characteristics $\mathbf{x}_0$ and $\hat{p}_0(\mathbf{x}_0)$ is the imputed log price for the same house in the base quarter 0. The price function $\hat{p}_t(\cdot)$ is the result of the rolling window regressions described in

Section 3.1 of the main paper. The continuous characteristics in the vector $\mathbf{x}_0$ correspond to the average quality of a detached house sold in 2011—the middle of our sample period—as reported in Gutachterausschuss für Grundstückswerte (2011). The discrete characteristics are set to the modal values of a detached house sold in that year (2011). We choose the location coordinates to represent an area in the south of Berlin—at the intersection of the districts Steglitz, Tempelhof, and Neukölln—that has the highest number of sales in that year (2011). Table C1 summarises $\mathbf{x}_0$.

[Table C1 about here.]

D Robustness checks

Sensitivity of the distribution analysis. We estimate $F_{j|k}(p)$ and the markup decomposition with a linear instead of a polynomial specification for the quantile regressions. Linear specifications are used frequently in empirical applications, as they are often less prone to omitted variables bias than more flexible specifications (Cropper et al. 1988, Kuminoff et al. 2010). Shimizu et al. (2016), in particular, use a linear specification in their analysis of ask and sale data distributions.

Figure D1 shows Q-Q plots for the empirical price distribution (EDF) and $\hat{F}_{j|k}(p)$, the latter estimated with a linear specification for the quantile regressions. The Q-Q plots are similar to those in Figure 4 of the main paper, where $\hat{F}_{j|k}(p)$ is estimated with a polynomial specification for the quantile regressions.

[Figure D1 about here.]

Table D1 reports the markup decomposition when the quantile regressions have a linear specification. The magnitudes of estimated markups and contributions of characteristics and implicit prices at the different quantiles are similar to those reported in Table 4 in the main paper.

[Table D1 about here.]

However, different to the results reported in the main paper, the contribution of implicit prices is always significant the 5% level. The decomposition results reported in the main paper are thus conservative.

WTP estimated for subperiods. The individual WTP in the main paper are computed from the hedonic regression fitted to the full sample. This assumes that the hedonic pricing function is stable over time. To examine whether this is sensible, we estimate the hedonic regressions of Eq. 6 in the main paper separately for non-overlapping subperiod partitions of the full sample: 2007-08, 2009-10, 2011-12, and 2013-15. Once estimated, we compute the individual WTP for the observations in the partition using the formulas for the summands in Eq. 9 and Eq. 10. This leads to N individual WTP estimates in total.

[Table D2 about here.]

Panel A of Table D2 reports the resulting WTP estimates for house characteristics. The WTP point estimates are similar to those in Table 5 in the main paper. The WTP for age remains statistically insignificant when estimated with the ask data. This—implausible—result is thus not an artefact of the choice of sample. The ratios of the WTP estimates from ask and sale data are

1.53 for the floor and 1.49 for the plot area when subperiods are used, only slightly different from 1.58 and 1.47 when the full sample is used. The ratios of the WTP estimated with ask to those estimated with sale data for each of the subperiods are similar to the WTP ratios for the floor and the plot area from Table 5 in the main paper. The main difference between Table D2 and Table 5 in the main paper are the much higher standard errors in the former, which indicates the price that has to be paid for more flexibility.

Panel B of Table D2 reports the WTP estimates of the noise level when the individual WTP is estimated separately for subperiods. The resulting WTP estimates are again similar to those reported in the main paper, see Table 6. However, the estimation over subperiods comes with much higher standard errors.

Imputation of exterior floor area. In the main paper, we use a constant conversion factor to obtain the exterior floor area (FA) from the interior area (IA) for observations in the ask data. A constant factor *could* introduce measurement error. To examine this, we use a different imputation method and fit

$$\text{(D1)} \qquad \ln\left(\frac{FA}{IA}\right) = \gamma_0 + \gamma_1 SEMI + \gamma_2 TERRACED + \gamma_3 AGE + \varepsilon$$

The regression relates the log ratio of the two areas to building characteristics that are observed in both data sets, which is necessary for the imputation. We fit Eq. D1 to a sample of 1,513 observations in the sale data that report both area characteristics. Table D3 gives summary statistics for this sample. Compared to the full sample—see Table 2 in the main paper—the sample is selective, as the observations have buildings that are on average younger, smaller, and more often semi-detached.

[Table D3 about here.]

Table D4 shows that the fit of the regression has a very small explanatory power and that age is the only characteristic that has a statistically significant coefficient estimate.

[Table D4 about here.]

At the average age among all sales, the estimated conversion function results in a factor of 1.11, smaller than the constant conversion factor of 1.25 that is suggested by the GAA. If we look at the correlations between the individual imputations and the actual *FA*, however, then these are nearly identical: $\hat{\rho} = 0.715$ for the estimated conversion function and $\hat{\rho} = 0.714$ for the constant conversion factor. This does not indicate that the constant conversion factor is inferior. Indeed, the correlation between the individual imputations conducted with the two different methods is nearly perfect with $\hat{\rho} = 0.999$.

Automated valuation using a parametric model. We assess whether a parametric model for the hedonic regressions would improve the predictive accuracy of valuations compared to those of the semiparametric additive model of Eq. 6 from the main paper. We use

$$\text{(D2)}\quad p = \mathbf{z}\boldsymbol{\gamma} + f_1(AGE;\boldsymbol{\beta}_1) + f_2(FA;\boldsymbol{\beta}_2) + f_3(PA;\boldsymbol{\beta}_3) + f_4(LAT, LON;\boldsymbol{\beta}_4) + \varepsilon$$

in the rolling window regressions, where $f_j(\cdot)$ is a d_j'th degree polynomial in continuous variable j, $d_j \in \{1, 2 \ldots, 7\}$, and $\boldsymbol{\beta}_j$ is a vector of coefficients. The vector $\mathbf{z}$ collects again all dummy variables. All variables are defined as in the main paper. For each training sample, we select $\mathbf{d} = \{d_1, d_2, d_3, d_4\}$ with

$$\text{(D3)}\qquad \hat{\mathbf{d}} = \arg\max_{\mathbf{d}} \left[1 - \frac{\sum_{i=1}^{N} (p_i - \hat{p}_{-i})}{\sum_{i=1}^{N} (p_i - \bar{p})}\right]$$

where $\hat{p}_{-i}$ is the leave-one-out estimator for observation i. We calculate the predictive residuals $(p_i - \hat{p}_{-i})$ from the ordinary least squares residuals and diagonal elements of the hat matrix (Myers 1990, pp. 172-73).

Table D5 gives performance measures for the out-of-sample predictions from the parametric regressions fitted separately to ask and sale data. Ask data lead to prediction errors that are severely biased *and* significantly more dispersed than prediction errors from sale data. This corresponds to what we find in the main paper.

[Table D5 about here.]

Close comparison of Table D5 and Table 7 from the main paper shows that the performance of the parametric model is inferior, irrespective whether the loss function is the L1 or L2 norm. This is evidence that our semiparametric model approximates the (unknown) hedonic price function better than the parametric polynomial model.

E Software packages

We implement the stochastic dominance tests and quantile markup decomposition with the user-written `Stata` function `cdeco`. The code can be installed from `https://sites.google.com/site/blaisemelly/`. The site also provides `R` code for counterfactual quantile decomposition. To estimate the semiparamteric regression models we employ the `gam()` function from the `R` package `mgcv`, see `https://cran.r-project.org/web/packages/mgcv/index.html`. Wood (2017) provides an excellent introduction to generalized additive models and the `mgcv` package.

References

Chernozhukov, V., Fernandez-Val, I. and Mellie, B.: 2013, Inference on counterfactual distributions, *Econometrica* **81**, 2205–2268.

Cropper, M. L., Deck, L. B. and McConnel, K. E.: 1988, On the choice of functional form for hedonic price functions, *Review of Economic and Statistics* **70**, 668–675.

Gutachterausschuss für Grundstückswerte: 2011, *Bericht über den Berliner Grundstücksmarkt 2010/11*, Senatsverwaltung für Stadtentwicklung, Berlin. Kulturbuch-Verlag Berlin.

Gutachterausschuss für Grundstückswerte: 2012, *Bericht über den Berliner Grundstücksmarkt 2011/12*, Senatsverwaltung für Stadtentwicklung und Umwelt, Berlin. Kulturbuch-Verlag Berlin.

Gutachterausschuss für Grundstückswerte: 2013, *Bericht über den Berliner Grundstücksmarkt 2012/13*, Senatsverwaltung für Stadtentwicklung und Umwelt, Berlin. Kulturbuch-Verlag Berlin.

Gutachterausschuss für Grundstückswerte: 2014, *Bericht über den Berliner Grundstücksmarkt 2013/14*, Senatsverwaltung für Stadtentwicklung und Umwelt, Berlin.

Gutachterausschuss für Grundstückswerte: 2015, *Bericht über den Berliner Grundstücksmarkt 2014/15*, Senatsverwaltung für Stadtentwicklung und Umwelt, Berlin.

Kuminoff, N. V., Parmeter, C. F. and Pope, J. C.: 2010, Which hedonic models can we trust to recover the marginal willingness to pay for environmental

amenities?, *Journal of Environmental Economics and Management* **60**, 145–160.

Myers, R. H.: 1990, *Classical and Modern Regression with Applications*, Duxbury Press, Belmont, California.

Shimizu, C., Nishimura, K. G. and Watanabe, T.: 2016, House prices at different stages of the buying/selling process, *Regional Science and Urban Economics* **59**, 37–53.

Wood, S. N.: 2003, Thin plate regression splines, *Journal of the Royal Statistical Society: Series B (Statistical Methodology)* **65**, 95–114.

Wood, S. N.: 2017, *Generalized additive models. An introduction with R*, Texts in Statistical Science, 2 edn, CRC Press, Boca Raton.

Wood, S. N. and Augustin, N. H.: 2002, GAMs with integrated model selection using penalized regression splines and applications to environmental modelling, *Ecological Modelling* **157**, 157–177.

Table A1: Bounds for data cleaning. Reports lower and upper bounds used for data cleaning procedure. Floor and plot area are in sqm. Source: Gutachterausschuss (2011, 2012, 2013, 2014, 2015).

Detached	Plot area		Floor area		Price per sqm	
West, vintage						
< 1949	400	1500	50	650	650	3530
≥ 1949	400	1500	50	625	780	2990
East, vintage						
< 1949	400	1500	50	510	410	2630
≥ 1949	400	1495	50	440	910	3185
Semi-detached						
West, vintage						
< 1949	215	700	80	455	665	3655
≥ 1949	175	700	65	360	1005	3055
East, vintage						
< 1949	230	700	40	330	430	2790
≥ 1949	190	700	60	210	1005	3350
Terraced houses						
West, vintage						
< 1949	130	695	65	470	720	3512
≥ 1949	115	700	75	335	895	3160
East, vintage						
< 1949	115	695	60	285	495	2085
≥ 1949	100	665	65	285	1095	2695

Table C1: Characteristics of reference house. Reports characteristics of reference house used to impute hedonic price index. Floor and plot area are in sqm. Latitude and longitude are the Universal Transverse Mercator (UTM) coordinates for the subway station U-Bahnhof Alt-Mariendorf.

Panel A. Continuous characteristics	
Age	20
Floor area	175
Plot area	650
Latitude	5810732
Longitude	392131.1
Panel B. Discrete characteristics	
Listed building	No
Prefabricated	No
Converted attic	No
Swimming pool	No
Flat roof	No
No basement	No
Backland development	No
Lake/River access	No
Condition of building	Average
Neighborhood amenity rating	Average
Buyer	Private person
Seller	Private person

Table D1: Alternative decomposition of markups. Shows decomposition of the ask and sale price distributions when a linear model is used for the quantile regressions of p on the core characteristics. Standard errors for the mean (quantile) decomposition are computed using the Huber-White covariance estimator (bootstrapped interquartile range of $\hat{F}_{j|k}(p)$). Pointwise confidence intervals use critical values from $N(0,1)$. Uniform confidence bands use empirical quantile of bootstrapped KS maximal t-statistic, see Chernozhukov et al. (2013, p. 2222). The number of bootstrap replications is 200. The confidence level is set to 0.95

	Estimated Effect	Standard Error	Pointwise Conf. Interv.		Uniform Conf. Bands	
Panel A. Markup						
Mean	0.234	0.004	0.225	0.242		
Quantile						
0.1	0.262	0.005	0.252	0.273	0.248	0.277
0.2	0.219	0.004	0.210	0.227	0.208	0.230
0.3	0.196	0.004	0.189	0.204	0.186	0.207
0.4	0.184	0.004	0.176	0.192	0.174	0.194
0.5	0.178	0.004	0.170	0.185	0.167	0.188
0.6	0.178	0.004	0.169	0.186	0.167	0.189
0.7	0.187	0.005	0.177	0.196	0.174	0.199
0.8	0.211	0.006	0.200	0.222	0.196	0.226
0.9	0.276	0.008	0.260	0.291	0.255	0.296
Panel B. Characteristics						
Mean	0.219	0.005	0.210	0.228		
Quantile						
0.1	0.169	0.003	0.162	0.176	0.160	0.177
0.2	0.150	0.003	0.144	0.156	0.142	0.157
0.3	0.139	0.003	0.134	0.145	0.132	0.147
0.4	0.135	0.003	0.129	0.140	0.128	0.142
0.5	0.133	0.003	0.127	0.139	0.126	0.140
0.6	0.138	0.003	0.132	0.144	0.130	0.146
0.7	0.152	0.004	0.145	0.159	0.143	0.161
0.8	0.180	0.004	0.172	0.189	0.169	0.191
0.9	0.250	0.006	0.238	0.261	0.235	0.265
Panel C. Implicit prices						
Mean	0.015	0.004	0.007	0.0228		
Quantile						
0.1	0.093	0.005	0.083	0.104	0.079	0.108
0.2	0.069	0.004	0.061	0.077	0.059	0.080
0.3	0.057	0.003	0.050	0.063	0.048	0.066
0.4	0.049	0.003	0.043	0.055	0.041	0.057
0.5	0.044	0.003	0.039	0.050	0.037	0.052
0.6	0.040	0.003	0.034	0.046	0.032	0.048
0.7	0.035	0.003	0.029	0.041	0.026	0.044
0.8	0.031	0.004	0.023	0.038	0.020	0.041
0.9	0.026	0.005	0.016	0.036	0.012	0.039

Table D2: Willingness to pay based on subperiod estimates. Panel A reports WTP estimates computed with Eq. 9 and Eq. 10 from the main paper, but with the functions in the summands estimated separately for partitions of the full sample. Specifications are identical to (2) and (5) from Table 5 in the main paper. Panel B reports WTP estimates for noise levels based on regression with $f_5(NOI)$ added. Standard errors are computed as the observation-weighted average of the bootstrap standard errors in each subperiod. Number of bootstrap replications in each subperiod is 200. Significant at ***0.001 level, **0.01 level, *0.05 level.

Panel A. House characteristics				
	Ask data		Sale data	
	WTP	Std. Err.	WTP	Std. Err.
Age	47.48	113.31	-1361.47***	261.69
Floor area	1287.22***	16.22	842.28***	30.98
Plot area	1379.80***	15.78	951.03***	30.41
Detached	18359.22***	2968.34	6718.59	5984.40
Semi-detached	7034.18**	2346.98	3498.83	4328.99
Listed			18733.87*	8324.88
Prefabricated			-5960.84	3467.39
Converted attic			3537.06	2501.85
Swimming pool			13633.14	10015.06
Flat roof			-5511.85	3277.10
No basement			-22722.32***	3083.81
Backland develop.			-586.62	2713.06
Waterfront			65806.10***	13594.82
Poor condition			-55056.85***	4747.57
Good condition			29720.73***	3279.17
Poor amenities			-8146.89**	2992.08
Good amenities			22256.35***	4873.70
Excel. amenities			43445.20*	18400.96
Panel B. Noise levels				
Noise levels	-1201.20***	169.40	-837.07***	220.69
$f_4(LAT, LON)$		Yes		Yes
Buyer/Seller dummies		No		Yes
Quarterly time dummies		Yes		Yes
N		59,502		12,218

Table D3: Summary statistics for observations in sale data that report floor and interior area. Number of observations is 1,513. Age of building at the date of sale. Areas are in sqm.

	Mean	Std. Dev.	Min	Max
Age	22.53	29.43	0.00	100.00
Area				
floor	141.76	46.29	45.00	451.00
interior	125.15	35.13	42.00	552.00
Detached	0.40			
Semi-detached	0.39			
Terraced house	0.19			

Table D4: Regression for area ratio. Shows OLS estimates of Eq. D1. Standard errors are computed using the heteroscedasticity robust Huber-White covariance estimator. Significant at ***0.001 level, **0.01 level, *0.05 level.

	Coef.	Std. Err.
Age	0.001*	0.000
Semi-detached	0.022	0.015
Terraced house	0.019	0.016
Constant	0.083***	0.013
R^2		0.004
N		1,513

Table D5: Assessment of prediction errors from parametric hedonic regression model. Shows performance statistics for 9,152 out-of-sample prediction errors. $\pm 10\%$ ($\pm 25\%$) reports the proportion of errors which are in absolute terms no larger than 10% (25%).

Data	MSE	Bias	Var.	Med.	MAE	$\pm 10\%$	$\pm 25\%$
Ask	0.089	-0.024	0.088	-0.012	0.231	0.282	0.610
Sale	0.059	0.009	0.059	0.021	0.189	0.335	0.697

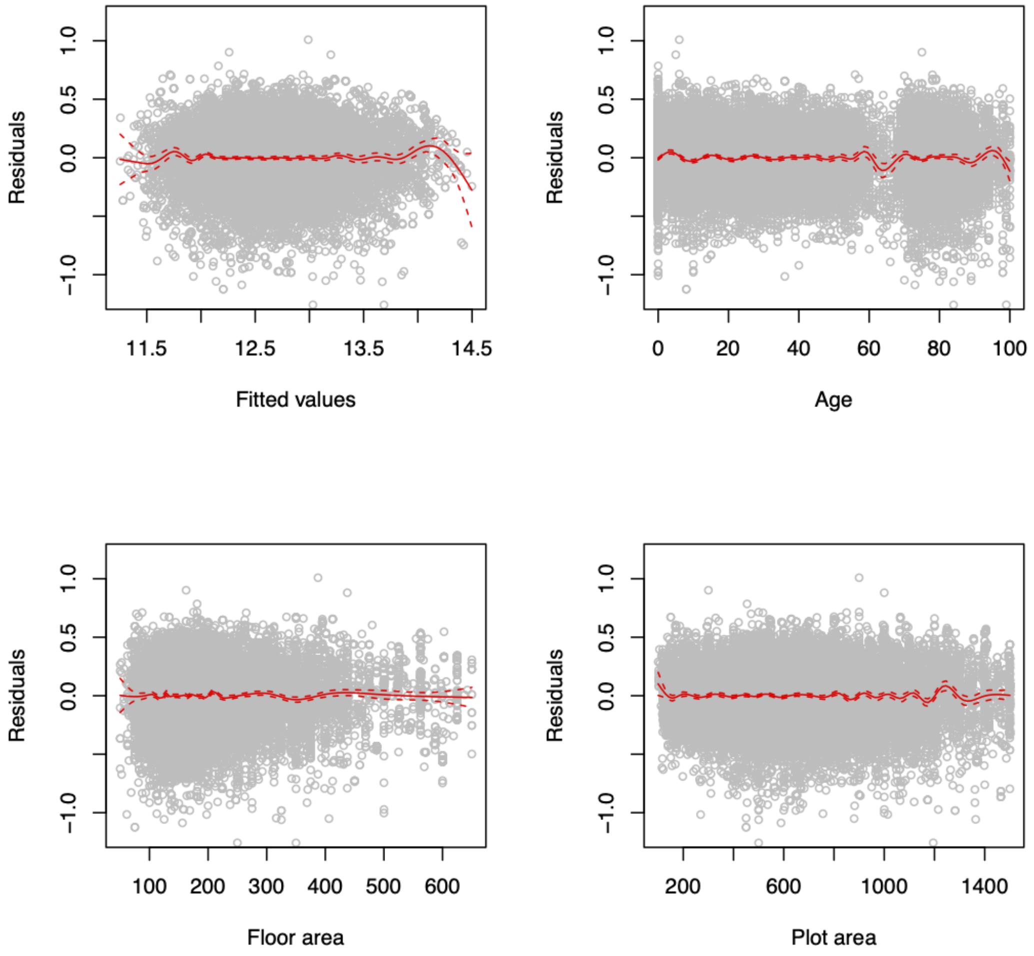

Figure B1: Residual diagnostics for ask data. Residuals come from specification (2) in Table 5 of the main paper. Solid red line is fitted cubic regression spline. in the ask price regression Dotted red lines are 0.95 pointwise confidence intervals.

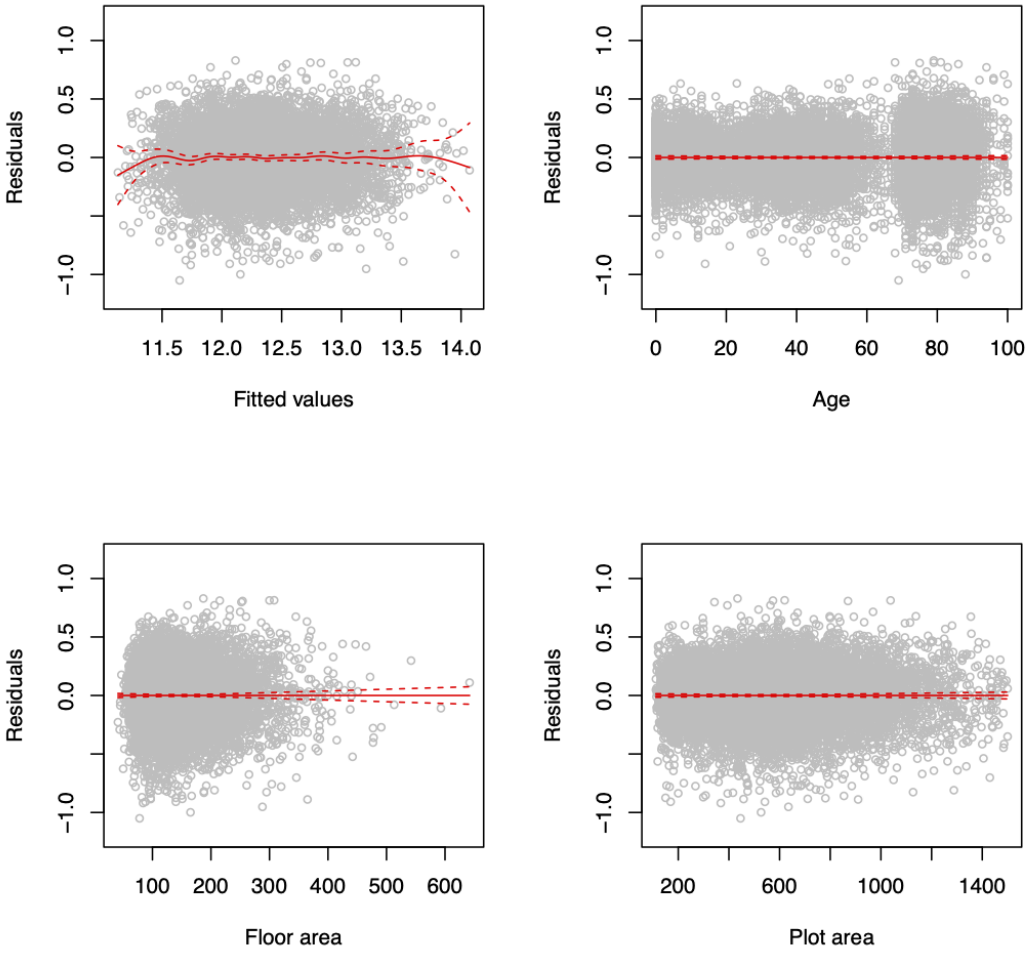

Figure B2: Residual diagnostics for sale data. Residuals come from specification (5) in Table 5 of the main paper. Solid red line is fitted cubic regression spline. The number of knots is set to $K = 29$. Dotted red lines are 0.95 pointwise confidence intervals.

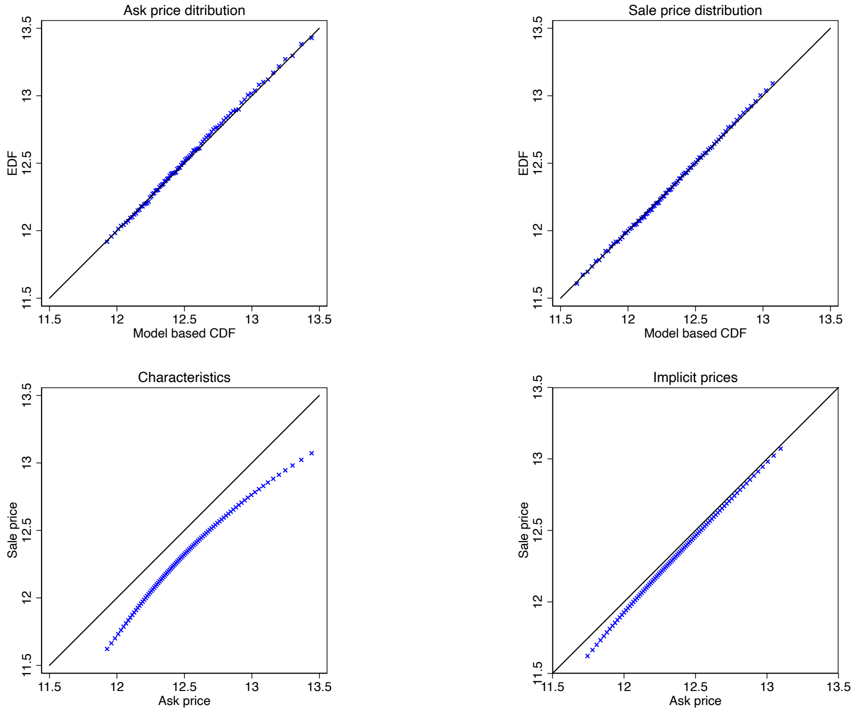

Figure D1: Q-Q plots for price distributions, linear specification. Upper-left (right) panel compares $\hat{F}_{a|a}$ ($\hat{F}_{s|s}$) to the EDF of the ask (sale) price, where $\hat{F}_{j|k}$ is estimated from Eq. 4 in the main paper. Lower-left (right) panel compares $\hat{F}_{a|a}$ ($\hat{F}_{a|s}$) to $\hat{F}_{a|s}$ ($\hat{F}_{s|s}$). Solid black line is the 45 degree line
